# Overcoming Gastric Barriers for Oral Peptide Delivery: QbD-Based Development of Sodium Caprate-Enabled Tirzepatide Tablets

**DOI:** 10.3390/pharmaceutics18070826

**Published:** 2026-07-05

**Authors:** Seokhyun Im, Ji-Yoon Lee, Joo-Eun Kim

**Affiliations:** 1 Department of Biopharmaceutical Chemistry, Kookmin University, Seoul 02707, Republic of Korea; ish970106@kookmin.ac.kr (S.I.); 6941as@kookmin.ac.kr (J.-Y.L.); 2 Department of Biopharmaceutical Chemistry, School of Applied Chemistry, Kookmin University, Seoul 02707, Republic of Korea; 3 Department of Pharmaceutical Engineering, Kookmin University, Seoul 02707, Republic of Korea

**Keywords:** tirzepatide, oral peptide delivery, sodium caprate, absorption enhancer, quality by design, dissolution, pharmacokinetics

## Abstract

**Background/Objectives**: Tirzepatide is a dual GIP and GLP-1 receptor agonist indicated for the treatment of type 2 diabetes and obesity. Oral delivery of tirzepatide is limited by poor gastrointestinal permeability, pH-dependent solubility, and manufacturing challenges associated with high-dose absorption enhancers. **Methods**: This study developed an immediate-release oral tirzepatide tablet using a Quality by Design (QbD) approach. Sodium caprate (C10) was selected as the absorption enhancer based on acid-neutralizing capacity, Caco-2 permeability enhancement, and preliminary rat pharmacokinetic screening. Quality target product profile, critical quality attributes, preliminary hazard analysis, and failure mode and effects analysis identified binder/disintegrant ratios as critical material attributes and hammer milling conditions as critical process parameters. Face-centered central composite designs and multiple-response optimization (MRO) were applied to optimize dissolution, flowability, and tablet mechanical integrity. **Results**: The optimized binder/disintegrant composition produced benchmark-comparable dissolution profiles against oral semaglutide tablets in pH 1.2, 4.0, and 6.8 media, with *f*_2_ values exceeding 50 for both C10 300 mg and 500 mg formulations. The optimized process yielded tablets with low friability (0.58%) and acceptable flowability (Carr’s index, 24). In beagle dogs, the C10 300 mg formulation achieved higher systemic exposure than the C10 500 mg formulation, with a *C*_max_ of 46.49 ± 23.79 ng/mL and *AUC*_last_ of 1261.03 ± 690.44 h·ng/mL. **Conclusions**: These results support C10-mediated oral tirzepatide delivery and QbD-based optimization for oral peptide tablets.

## 1. Introduction

The global burden of obesity and type 2 diabetes mellitus (T2DM) continues to increase, posing a substantial public health challenge [1]. According to recent World Health Organization estimates, 43% of adults worldwide were overweight in 2022, and 16% were living with obesity [2]. Obesity is one of the major modifiable risk factors for T2DM, with the lifetime risk of diabetes increasing markedly as body mass index (BMI) rises; in a U.S. population-based analysis, the estimated lifetime risk reached approximately 70% in men and 74% in women with severe obesity [3]. Given that T2DM is associated with long-term complications, including cardiovascular and renal diseases, and that lifestyle modification alone is often insufficient to achieve durable glycemic and metabolic control, there remains a strong clinical need for effective pharmacological strategies [4,5].

Glucagon-like peptide-1 (GLP-1) is an endogenous incretin hormone that regulates glucose homeostasis and body weight through multiple mechanisms, including glucose-dependent stimulation of insulin secretion, suppression of glucagon release, delayed gastric emptying, and reduced appetite [6]. Owing to these pleiotropic metabolic effects, the GLP-1 receptor has become a major therapeutic target for the treatment of T2DM and obesity. However, native GLP-1 has an extremely short circulating half-life of approximately 1–2 min because it is rapidly degraded by dipeptidyl peptidase-4 [7], which has led to the development of GLP-1 receptor agonists with improved enzymatic stability and prolonged pharmacological activity. More recently, therapeutic strategies have expanded beyond GLP-1 receptor mono-agonism toward multi-incretin receptor agonism, as exemplified by tirzepatide. Tirzepatide is a first-in-class, acylated peptide co-agonist engineered to activate both glucose-dependent insulinotropic polypeptide (GIP) and GLP-1 receptors [8]. Clinical studies have shown that tirzepatide provides greater reductions in HbA1c and body weight than GLP-1 receptor mono-agonists, including semaglutide; in obesity trials, tirzepatide has produced mean body weight reductions approaching 20–21% [9], with a recent head-to-head trial reporting a 20.2% reduction with tirzepatide versus 13.7% with semaglutide at 72 weeks [10].

Despite its well-established clinical efficacy, tirzepatide is currently available only as a subcutaneous (SC) injectable product. Although once-weekly dosing improves convenience compared with daily injections, injectable therapy can still be associated with injection-site discomfort, needle-related anxiety, and reduced patient acceptance, particularly in the context of chronic long-term treatment [11]. In addition, refrigerated storage requirements and the need for appropriate disposal of needles or injection devices may impose practical burdens on patients and healthcare systems [12]. Therefore, the development of a non-invasive oral dosage form of tirzepatide would be a valuable strategy to improve patient convenience, treatment acceptability, and accessibility of incretin-based therapy [13].

However, the oral delivery of tirzepatide remains highly challenging because it is a large synthetic peptide consisting of 39 amino acids with a molecular weight of approximately 4.8 kDa. Like other peptide therapeutics, tirzepatide is vulnerable to chemical and enzymatic degradation in the gastrointestinal tract, while its large molecular size, high polarity, and limited passive epithelial permeability severely restrict systemic absorption after oral administration [14]. The successful development of oral semaglutide with salcaprozate sodium (SNAC) has demonstrated that formulation-enabled peptide absorption is clinically feasible. SNAC promotes gastric absorption of semaglutide by locally increasing the pH around the tablet, thereby reducing proteolytic degradation and facilitating transcellular transport across the gastric epithelium [15]. Inspired by this absorption-enhancing strategy, the present study investigated sodium caprate (C10) [16], a medium-chain fatty acid salt, as a functional excipient for oral tirzepatide delivery. C10 has been reported to enhance epithelial permeability through multiple, potentially interrelated mechanisms, including modulation of tight junctions and perturbation of the epithelial plasma membrane [17]. Mechanistically, C10 acts as a mild non-ionic surfactant that enhances epithelial permeability through dual pathways. For paracellular transport, C10 activates phospholipase C, leading to an increase in intracellular inositol triphosphate (IP3) and Ca^2+^ levels. This cascade activates calmodulin and myosin light-chain kinase (MLCK), triggering the contraction of the perijunctional actomyosin ring and the reversible structural alteration of specific tight junction proteins, such as zonula occludens-1 (ZO-1) and claudins [17]. Simultaneously, for transcellular transport, C10 intercalates into the lipid bilayer of the epithelial membrane, promoting membrane fluidization and reducing barrier integrity without causing irreversible cytotoxicity [18]. Furthermore, the translational viability of C10 as an absorption enhancer is strongly supported by existing clinical data and pharmaceutical patents. Clinically, C10 has been successfully incorporated into oral formulations of large molecules, such as oral insulin (e.g., Tregopil) and antisense oligonucleotides, which have advanced into human clinical trials [19]. Additionally, the recent pharmaceutical literature highlights the extensive utilization of C10 and its derivatives specifically for the formulation of oral GLP-1 and dual GIP/GLP-1 receptor agonists, underscoring its translational relevance and efficacy in incretin-based oral delivery systems [13]. These well-documented, transient, and reversible permeation-enhancing effects make C10 a highly rational and validated candidate for this study [20]. Therefore, this study aimed to develop an oral tirzepatide tablet incorporating C10 and to evaluate whether this strategy could overcome the key stability and permeability barriers associated with oral peptide delivery.

In addition to biological barriers, the development of an oral tirzepatide tablet requires a robust manufacturing strategy capable of producing tablets with reproducible quality. Dry granulation is an attractive approach for peptide formulations that are sensitive to moisture and thermal stress [21]; however, powders containing a high proportion of C10 can exhibit poor flowability, cohesiveness, and compactability. In particular, an inappropriate particle size distribution, especially the excessive generation of fines during milling, may impair die filling and interparticulate bonding, thereby increasing the risk of mechanical defects such as lamination and high friability [22]. Conversely, excessive binder incorporation to improve tablet strength may delay disintegration and compromise rapid drug release, which is necessary to generate a high local concentration of both tirzepatide and the absorption enhancer at the absorption site [23]. Therefore, formulation variables and process parameters should be optimized in an integrated manner to achieve the desired critical quality attributes (CQAs), including target dissolution behavior and mechanical integrity [24].

Accordingly, the objective of this study was to develop and evaluate a C10-based immediate-release oral tablet of tirzepatide using a Quality by Design (QbD) approach [25,26,27,28,29]. Prior to formulation optimization, preformulation studies were performed to characterize the physicochemical properties of tirzepatide and to assess its compatibility with selected excipients. C10 was incorporated as a functional excipient based on its acid-neutralizing capacity and permeation-enhancing potential. Its effective level was investigated through in vitro Caco-2 permeability studies and preliminary in vivo pharmacokinetic (PK) screening in rats [30]. Subsequently, a QbD framework was applied to establish a robust formulation and manufacturing process. Based on the Quality Target Product Profile (QTPP), CQAs were defined, and potential critical material attributes (CMAs) and critical process parameters (CPPs) were identified through preliminary hazard analysis (PHA) and failure mode and effects analysis (FMEA) [31]. These variables were then systematically optimized using a Design of Experiments (DoE) approach to define an acceptable design space. Finally, the optimized formulation was evaluated through in vitro dissolution testing and in vivo PK assessment in beagle dogs [32] to determine whether the developed oral tirzepatide tablet could enhance systemic exposure and support the translational potential of C10-mediated peptide delivery.

## 2. Materials and Methods

### 2.1. Materials

Tirzepatide (purity ≥ 99.5%) was purchased from Fujian Genohope Biotech Ltd. (Putian, China). Lactose monohydrate and microcrystalline cellulose were obtained from DFE Pharma (Goch, Germany). Povidone K25 (K25) and povidone K90 (K90) were purchased from Ashland Inc. (Wilmington, DE, USA), and croscarmellose sodium was purchased from IFF (Wilmington, DE, USA). Copovidone VA64 (VA64) and crospovidone (CROS) were obtained from BASF SE (Ludwigshafen, Germany). Sodium starch glycolate (SSG) and mannitol were purchased from Roquette Frères (Lestrem, France). C10 (purity ≥ 99.0%) and SNAC (purity ≥ 98.0%) were obtained from Tokyo Chemical Industry Co., Ltd. (Tokyo, Japan) and Cayman Chemical (Ann Arbor, MI, USA), respectively. Magnesium stearate was purchased from Nitika Pharmaceutical Specialties Pvt. Ltd. (Nagpur, India). Sodium bicarbonate and talc were obtained from Hebei Huachen Pharmaceutical Co., Ltd. (Cangzhou, China). High-performance liquid chromatography (HPLC)-grade acetonitrile, ethanol, and methanol were obtained from Duksan Pure Chemicals Co., Ltd. (Ansan, Republic of Korea). Ultrapure water with a resistivity of 18.2 MΩ·cm was prepared using an in-house water purification system. All other chemicals and reagents were of analytical grade and used as received. Hank’s balanced salt solution (HBSS) and 4-(2-hydroxyethyl)-1-piperazineethanesulfonic acid (HEPES) buffer solution were obtained from Welgene Inc. (Gyeongsan, Republic of Korea). Hydrochloric acid and Tween 80 were purchased from Daejung Chemicals & Metals Co., Ltd. (Siheung, Republic of Korea). 3-(4,5-dimethylthiazol-2-yl)-2,5-diphenyltetrazolium bromide (MTT) reagent, 3-(4,5-dimethylthiazol-2-yl)-2,5-diphenyltetrazolium bromide, was purchased from Sigma-Aldrich (St. Louis, MO, USA). The Lactate dehydrogenase (LDH) cytotoxicity assay was performed using a Cytotoxicity LDH Assay Kit-WST purchased from Dojindo Molecular Technologies, Inc. (Kumamoto, Japan). All other excipients used in the formulations were of pharmaceutical grade. All materials were employed throughout the experiments without further purification.

### 2.2. Preformulation and Absorption Enhancer Screening

Prior to formulation optimization, preformulation studies were conducted to characterize the physicochemical properties of tirzepatide and to establish the scientific basis for rational formulation design. These studies included solubility profiling, drug–excipient compatibility assessment, and functional evaluation of absorption enhancers. The absorption-enhancing performance of the candidate enhancer was evaluated using in vitro Caco-2 cell permeability assays and preliminary in vivo PK studies in rats; the obtained results subsequently provided a data-driven rationale for formulation design and optimization. While the overall experimental frameworks were adapted from established literature and pharmacopeial guidelines, the specific analytical procedures, including the HPLC methods for drug quantification and initial dose-setting strategies, were developed and validated in-house.

#### 2.2.1. Morphology

The particle morphology of tirzepatide was examined by scanning electron microscopy (SEM; JSM-7401F, JEOL Ltd., Tokyo, Japan). The powder sample was placed on an aluminum specimen stub covered with double-sided conductive carbon tape and coated with a thin layer of platinum using a sputter coater (208HR, Cressington Scientific Instruments Ltd., Watford, UK) for 1 min under vacuum. SEM images were acquired at an accelerating voltage of 5 kV at different magnifications. For image-based particle size estimation, at least 100 individual particles were randomly selected from representative micrographs and analyzed using ImageJ software (version 1.54g, National Institutes of Health, Bethesda, MD, USA) [33].

#### 2.2.2. RP-HPLC Analytical Method

Samples were filtered through a 0.45 μm polytetrafluoroethylene (PTFE) membrane filter and analyzed by HPLC using an Agilent 1260 Infinity II LC system (Agilent Technologies, Santa Clara, CA, USA) equipped with a UV detector set at 220 nm. Chromatographic separation was performed on an Agilent C18 column (4.6 × 150 mm, 5 μm, Agilent Technologies, Santa Clara, CA, USA) maintained at 30 °C. The mobile phase consisted of 0.05% (*v*/*v*) formic acid (FA) in ultrapure water (solvent A) and 0.05% (*v*/*v*) FA in ace-tonitrile (solvent B). The flow rate and injection volume were set at 1.0 mL/min and 20 μL, respectively. Gradient elution was performed as follows: 10% B at 0 min, a linear increase to 90% B over 20 min, a decrease to 10% B at 20.1 min, and maintenance at 10% B until 25 min for column re-equilibration. Data were acquired and processed using Agilent OpenLab CDS software (version 2.6). System suitability, calibration linearity, and method specificity were confirmed before sample quantification. Under these chromatographic conditions, the retention time of tirzepatide was approximately 12.2 min.

#### 2.2.3. Measurement of Tirzepatide Solubility

The solubility profile of tirzepatide, including apparent clear solubility and equilibrium solubility, was evaluated in accordance with United States Pharmacopeia (USP) <1236> [34]. The tested media included distilled water, representative organic solvents (ethanol, methanol, and acetonitrile), and aqueous buffer solutions covering a pH range of 1.2–12.0. The solubility in these organic solvents was specifically determined to establish optimal solvent and mobile phase conditions for the subsequent HPLC and LC-MS/MS analytical methods. Given the high cost of the peptide, the apparent clear solubility was initially measured as a rapid screening tool to conserve the active pharmaceutical ingredient (API) and to accurately estimate the required excess amount for the definitive equilibrium solubility test. For apparent clear solubility determination, 10 mg of tirzepatide was accurately weighed, and each solvent was added stepwise in 500 μL increments under continuous stirring at 400 rpm and 25 °C until no visible undissolved particles remained. The solvent volume required for apparent dissolution was used to assign the descriptive USP solubility classification.

Based on the initial solubility screening, equilibrium solubility was further determined by adding excess tirzepatide to each selected medium. The suspensions were stirred at 400 rpm and 15 °C, and samples were collected at 0, 2, 12, and 24 h to confirm that equilibrium had been reached. Each aliquot was filtered through a 0.45 μm PTFE syringe filter to remove undissolved drug particles, diluted as necessary, and analyzed using a HPLC method to quantify the dissolved tirzepatide concentration. To ensure accuracy, all equilibrium solubility measurements were performed in triplicate (*n* = 3).

#### 2.2.4. Drug–Excipient Compatibility Studies

Drug–excipient compatibility studies were performed to identify potential physicochemical interactions between tirzepatide and selected excipients during formulation development. Binary mixtures of tirzepatide and each excipient were prepared at a 1:1 weight ratio and uniformly blended [35]. The mixtures were placed in glass vials and stored at 4 ± 2 °C and 25 ± 2 °C/60 ± 5% RH for 4 weeks. Tirzepatide alone was stored under the same conditions to monitor the intrinsic stability of the drug in the absence of excipients. At predetermined time points of 0, 2, and 4 weeks, the samples were visually inspected for any visible changes, including discoloration, aggregation, or deliquescence. For compatibility assessment, each sample was dissolved in methanol, filtered through a 0.45 μm PTFE syringe filter, and analyzed using the validated HPLC method.

#### 2.2.5. In Vitro Assessment of the Acid-Neutralizing Capacity of C10

The acid-neutralizing capacity and dispersion behavior of C10 were evaluated using volume-adjusted acidic media designed to represent simplified fasting gastric conditions in beagle dogs and humans [36,37]. The beagle dog gastric model consisted of 25 mL of 0.01 N HCl, representing the basal fasting gastric fluid volume characteristic of this animal model, supplemented with 10 mL of purified water to simulate the co-administered water volume during dosing [15]. In contrast, the human gastric model consisted of 50 mL of 0.01 N HCl supplemented with 120 mL of purified water, simulating the co-administered water volume in accordance with the dosing guidelines of a benchmark oral peptide therapy [38]. C10 at doses of 100, 300, and 500 mg was added to each medium in a 200 mL beaker and dispersed under continuous magnetic stirring at 150 rpm at room temperature. The pH of each dispersion was measured using an OHAUS Starter 3100 pH meter (OHAUS Corporation, Parsippany, NJ, USA) at 15 and 45 min to evaluate the initial and time-dependent acid-neutralizing effects of C10.

#### 2.2.6. Screening of Absorption Enhancers in Caco-2 Cell Monolayers

Caco-2 cell monolayers were cultured on 12-well Transwell^®^ polyethylene terephthalate (PET) inserts with a pore size of 0.4 μm (Corning Inc., Corning, NY, USA). The apical transport medium consisted of HBSS buffered with 25 mM HEPES at pH 7.4, whereas the basolateral medium consisted of the same buffer supplemented with 0.02% (*w*/*v*) Tween 80 to minimize non-specific adsorption of peptide drugs [39]. Prior to the assay, monolayer integrity was confirmed by measuring transepithelial electrical resistance (TEER) [40]. The monolayers were washed twice with pre-warmed HBSS in both the apical and basolateral chambers and equilibrated at 37 °C for 15 min. Subsequently, 0.5 mL of donor solution containing 200 μM peptide drug with varying concentrations of C10 or SNAC was added to the apical chamber, and 1.5 mL of fresh transport medium was added to the basolateral chamber. After incubation at 37 °C under 5% CO_2_ for 1 h, samples were collected from both the apical and basolateral chambers, centrifuged, and analyzed using a validated HPLC method. The apparent permeability coefficient (*P*_app_) was calculated using the following equation [30]:

$$P_{app}=\frac{dQ/dt}{\left(A\cdot C_{0}\right)}$$

where *dQ*/*dt* is the steady-state permeation rate (μM/s), A is the effective membrane surface area (cm^2^), and *C*_0_ is the initial apical donor concentration (μM).

#### 2.2.7. In Vivo Pharmacokinetic (PK) Screening in Rats

All animal experiments were approved by the Institutional Animal Care and Use Committee (IACUC) of the affiliated institution (Approval No. 202507-01; approved on 3 July 2025) and conducted in accordance with institutional guidelines for the care and use of laboratory animals. Male Sprague–Dawley rats (8 weeks old, 250–300 g; total *n* = 36) were obtained from Young Bio (Seongnam, Republic of Korea). The animals were housed under environmentally controlled conditions and fasted overnight for 12 h with free access to water before dosing. The rats were randomly assigned to six groups (*n* = 6 per group): vehicle control, tirzepatide alone, tirzepatide with C10 at 100, 200, or 300 mg/kg, and tirzepatide with SNAC at 200 mg/kg. Tirzepatide was administered at a dose of 3 mg/kg in all tirzepatide-treated groups. All test formulations were administered once by oral gavage at a dosing volume of 10 mL/kg.

Serial blood samples of approximately 350 μL were collected from the jugular vein into K_2_EDTA tubes at pre-dose and at 0.5, 1, 2, 4, 6, 8, and 24 h after dosing [41]. Plasma was separated by centrifugation at 12,000 rpm for 5 min at 4 °C and stored at −80 °C until analysis.

### 2.3. QbD-Based Formulation and Process Optimization

A systematic QbD approach was applied to optimize the formulation and manufacturing process of the oral tirzepatide tablet in accordance with ICH Q8(R2) principles [42]. The development process began with the establishment of the QTPP, followed by the identification of CQAs relevant to product performance, safety, and quality. Initial risk assessments were performed to identify and prioritize formulation variables and process parameters with potential effects on the predefined CQAs. Subsequently, an FCCD was employed to evaluate the linear, interaction, and quadratic effects of the selected CMAs and CPPs [24]. The resulting DoE framework was used to support the simultaneous optimization of formulation and process variables and to define an acceptable design space for the final tablet formulation.

#### 2.3.1. QTPP, CQAs, and Risk Assessment

In accordance with the QbD principles described in ICH Q8(R2) [42], the QTPP was established at an early stage to guide the development of the oral tirzepatide tablet. Because no marketed oral tirzepatide product was available at the time of this study, oral semaglutide tablets were used as a clinically established benchmark for oral peptide delivery [13]. Accordingly, the target product was defined as a 14 mg immediate-release oral tablet designed to achieve systemic exposure through gastrointestinal absorption. Based on this QTPP, CQAs relevant to product quality, performance, and safety were identified. Assay, content uniformity, impurity profile, dissolution behavior, and mechanical integrity were selected as key CQAs for subsequent formulation and process optimization [43].

Given the specific formulation goals and processing constraints, dissolution behavior and mechanical integrity were prioritized as the primary CQAs for formulation and process optimization. For oral peptide delivery, rapid and reproducible dissolution is essential for generating sufficient local concentrations of tirzepatide and the absorption enhancer at the absorption site [23]. Therefore, the dissolution profile of the developed formulation was designed to approximate the benchmark oral peptide tablet profile while maintaining immediate-release characteristics (Table 1). In parallel, sufficient mechanical integrity was required to ensure manufacturability and dosage-form robustness. This was particularly important for C10-rich formulations, as high C10 content can impair powder flow, compactability, and interparticulate bonding, thereby increasing the risk of lamination and friability. Since particle size distribution and granule quality are strongly influenced by the sizing process, milling parameters and subsequent tableting conditions were considered key CPPs requiring systematic optimization.

A two-step risk assessment framework was applied to identify formulation and process variables with potential impacts on the predefined CQAs. First, PHA was performed based on prior scientific knowledge and preliminary experimental observations to identify potential risks associated with material attributes, process parameters, equipment-related factors, and environmental conditions. Subsequently, failure mode and effects analysis (FMEA) was used to further prioritize the identified risks [24]. The occurrence (O), severity (S), and detectability (D) of each potential failure mode were scored on a scale of 1 to 5, where higher scores indicated a higher likelihood of occurrence, greater severity, and lower detectability, respectively. The risk priority number (RPN) was calculated as O × S × D, and variables with an RPN greater than 30 were classified as high-risk factors. High-risk variables were then either selected for subsequent DoE optimization or assigned appropriate control strategies, depending on their controllability and relevance to formulation and process development.

#### 2.3.2. Optimization Design for CMAs

Based on the preliminary screening results and FMEA outcomes, the initial formulation was designed to contain tirzepatide and a high amount of C10 to address the key stability and permeability barriers associated with oral peptide delivery. The tirzepatide dose was fixed at 14 mg, which was selected with reference to the highest marketed strength of the benchmark drug, Rybelsus [44]. For oral peptide formulations containing a high level of permeation enhancer, rapid and reproducible drug release is essential to promote local co-exposure of the peptide and enhancer at the absorption site before extensive luminal degradation occurs [12]. Therefore, the binder fraction (*X*_1_) and disintegrant fraction (*X*_2_), which strongly influence tablet disintegration and dissolution behavior, were selected as CMAs. As these variables were identified as high-risk factors in the risk assessment, they were used as independent variables for subsequent formulation optimization using a face-centered central composite design (FCCD). The base composition of the initial immediate-release formulation is summarized in Table 2.

#### 2.3.3. Optimization Design for CPPs

To minimize exposure of moisture- and heat-sensitive tirzepatide to aqueous or thermal stress while improving powder flow and compactability, a slugging-based dry granulation process was employed [21]. All intragranular components were accurately weighed and blended according to the DoE-defined formulation composition. The resulting powder blend was compacted into slugs using a rotary tablet press (PR-LM 08, PTK Co., Ltd., Gimpo, Republic of Korea). The slugs were then milled using a hammer mill (Polymix PX-MFC 90 D, Kinematica AG, Malters, Switzerland).

For the sizing process, milling speed (*X*_3_) and milling screen size (*X*_4_) were selected as CPPs because of their potential effects on granule size distribution, flowability, and compactability. Preliminary trials showed that excessively high milling speeds, such as 2000 rpm, generated unfavorable granule characteristics, including excessive fines and altered particle morphology [45]. These changes were associated with poor compactability and an increased risk of lamination in C10-rich formulations. Therefore, the upper limit of milling speed was set at 1000 rpm. The milling screen size was evaluated within the available equipment range of 1.0–3.0 mm. The levels and ranges of all independent variables used in the DoE are summarized in Table 3. After sizing, the granules were blended with magnesium stearate passed through a 35-mesh sieve and compressed into immediate-release tirzepatide tablets for subsequent evaluation. All processing parameters other than the DoE-defined variables were kept constant throughout the experiments to ensure that the observed differences in granule and tablet properties were attributable to the selected CPPs.

### 2.4. Evaluation of CQAs

To establish the relationship between the independent formulation variables and product quality, the predefined CQAs and processability-related responses were quantitatively evaluated as response variables (*Y*) for all experimental runs generated by the FCCD. The evaluations included the flowability of granules prior to compression, the friability of compressed tablets, and in vitro dissolution performance. After statistical optimization of the formulation variables, the same analytical procedures were applied to the optimized formulation to compare the observed response values with the model-predicted values [46].

#### 2.4.1. Evaluation of Granule Flowability

To evaluate the flowability-related properties of the pre-compression granules, bulk density (*D*_B_) and tapped density (*D*_T_) were measured. These parameters were used as processability indicators because they can affect powder flow, die filling, tablet weight, and content uniformity [47]. The measurements were performed with reference to the general procedures described in USP <616> and <1174>, as well as the Korean Pharmacopoeia (KP) [48,49,50].

Briefly, an accurately weighed granule sample was gently transferred into a 50 mL graduated cylinder, and the initial volume (*V*_0_) and sample mass (*M*) were recorded to calculate *D*_B_. The cylinder was then manually tapped, and the final volume (*V*_f_) was recorded after 100 taps, at which a constant volume had been confirmed in preliminary testing. All measurements were performed in triplicate. Carr’s index (CI) was calculated as follows [51]:

CI = 100 × (*D*_T_ − *D*_B_)/*D*_T_.

#### 2.4.2. Evaluation of Tablet Friability

Tablet friability was evaluated according to USP <1216> and the KP [52,53]. Because the individual tablet weight exceeded 650 mg, 10 intact tablets were randomly selected from each batch. The tablets were weighed before testing and placed in a friability tester (CS-2, Tianjin Jingtuo Instrument Technology Co., Ltd., Tianjin, China). The test was performed for 100 revolutions at 25 rpm for 4 min. After testing, the tablets were dedusted and reweighed. Friability was calculated as the percentage weight loss relative to the initial tablet weight. All measurements were performed in triplicate, and the results are expressed as mean ± standard deviation.

#### 2.4.3. In Vitro Dissolution Study

In vitro dissolution profiles of the immediate-release tirzepatide tablets were evaluated using Rybelsus, the only commercially available oral GLP-1 receptor agonist, as a benchmark drug. Dissolution tests were performed in accordance with USP <711> and the KP [54,55]. The tests were conducted using a 708-DS dissolution apparatus (Agilent Technologies, Santa Clara, CA, USA) equipped with USP Apparatus 2 (paddles). Each vessel contained 900 mL of dissolution medium at pH 1.2, 4.0, or 6.8, and the paddle speed and temperature were maintained at 50 rpm and 37.0 ± 0.5 °C, respectively. To improve tirzepatide solubilization under acidic conditions and maintain sink-like conditions, 0.75% polyoxyethylene lauryl ether (Brij35) was added to the pH 1.2 and pH 4.0 media, whereas the pH 6.8 medium was used without surfactant. Aliquots (4 mL) were withdrawn at predetermined time points (5, 10, 15, 20, 30, 45, and 60 min; *n* = 6 per condition), filtered through 0.45 μm PTFE syringe filters, and analyzed using the established HPLC method.

Dissolution profile similarity was evaluated using the similarity factor (*f*_2_) in accordance with the Ministry of Food and Drug Safety guideline [56]. Because only one time point after drug release exceeds 85% should be included in the *f*_2_ calculation, the time points used for analysis were selected separately for each dissolution medium. In the pH 1.2 medium, where the benchmark drug showed gradual release and exceeded 85% dissolution only at 60 min, five time points (10, 15, 30, 45, and 60 min) were included. In contrast, because both the pH 4.0 and pH 6.8 media showed rapid dissolution behavior, with release exceeding 85% by 30 min, three time points were used for *f*_2_ calculation: 10, 20, and 30 min for pH 4.0, and 10, 15, and 30 min for pH 6.8. Formulations with an *f*_2_ value of ≥50 were considered to have dissolution profiles comparable to that of the benchmark drug.

### 2.5. In Vivo Pharmacokinetic (PK) Study Design in Beagle Dogs

In vivo PK profiles were evaluated using healthy male Marshall Beagle dogs (Canis lupus familiaris; 7.7–10.6 kg, 20–29 months of age). The animals were acclimatized in a climate-controlled facility (21.4–23.1 °C, 49.1–60.4% relative humidity) under a 12 h light/dark cycle. All procedures complied strictly with international animal welfare guidelines and were approved by the IACUC of CentralBio Co., Ltd. (Incheon, Republic of Korea) (Approval No. CBIACUC_CE26-0018PA, approved on 27 February 2026). Prior to dosing, all subjects were fasted for approximately 16 h, with feeding reinstated 4 h post-dose; water was provided ad libitum.

The dogs were randomized into three experimental groups (*n* = 3 per group). Group 1 received a single intravenous (IV) injection of tirzepatide (0.5 mg/animal). Groups 2 and 3 received a single oral administration of the optimized tirzepatide tablets (14 mg/animal), co-formulated with 300 mg and 500 mg of C10, respectively. In addition, PK data from a Rybelsus-treated group (14 mg/animal) were included as a historical benchmark comparator. These data were obtained from a historical control group previously evaluated in our laboratory under identical experimental conditions, in accordance with the 3Rs principle (Replacement, Reduction, and Refinement) to minimize animal use [57,58].

Serial blood samples of approximately 1 mL were collected from the jugular vein into tubes containing sodium heparin. For the IV group, blood samples were collected at 0.083 h (5 min), 0.25, 0.5, 1, 2, 4, 8, 24, and 48 h post dose. For the oral tablet groups, samples were collected at 0.25, 0.5, 1, 2, 4, 8, 12, 24, and 48 h post dose. Plasma was separated immediately by centrifugation at 12,000 rpm for 2 min at 4 °C and stored below −70 °C until quantitative analysis.

### 2.6. UHPLC-MS/MS Bioanalysis of Plasma Samples

The same validated UHPLC-MS/MS method was applied for the quantification of tirzepatide in both rat and Beagle dog plasma samples, in accordance with international regulatory guidelines for bioanalytical method validation [59]. The analytical system consisted of an Agilent 1290 Infinity II LC system coupled to a Sciex Triple Quad 5500+ mass spectrometer (SCIEX, Framingham, MA, USA). For sample preparation, 30 μL of plasma was mixed with 10 μL of goserelin as an internal standard (IS, 200 ng/mL), followed by protein precipitation with 100 μL of 1% formic acid in acetonitrile. The mixture was vortex-mixed at 2000 rpm for 2 min and centrifuged at 15,000 rpm for 5 min at 4 °C. The resulting supernatant was collected and injected into the UHPLC-MS/MS system.

Chromatographic separation was achieved using a PC HILIC S3 column (2.0 × 100 mm, 3 μm; Osaka Soda, Osaka, Japan) maintained at 40 °C. The mobile phases consisted of 10 mM ammonium formate with 0.2% formic acid in ultrapure water (mobile phase A) and 0.2% formic acid in acetonitrile (mobile phase B). Gradient elution was performed at a flow rate of 0.45 mL/min. Detection was performed in positive electrospray ionization mode using multiple reaction monitoring (MRM). The monitored transitions were *m*/*z* 1204.0 > 396.2 for tirzepatide and *m*/*z* 635.5 > 607.4 for the IS.

### 2.7. Pharmacokinetic (PK) Analysis

PK parameters were calculated from the in vivo plasma concentration–time data by non-compartmental analysis using Phoenix WinNonlin software (version 8.7; Certara, Princeton, NJ, USA) [60]. The maximum plasma concentration (*C*_max_) and the time to reach *C*_max_ (*T*_max_) were obtained directly from the individual plasma concentration–time profiles. The area under the plasma concentration–time curve from time zero to the last measurable concentration (*AUC*_last_) was calculated using the linear trapezoidal method [61].

### 2.8. Statistical Analyses

All data are expressed as mean ± standard deviation (SD), unless otherwise stated. Statistical comparisons among treatment groups for in vitro evaluation parameters and PK parameters were performed using one-way analysis of variance (ANOVA), followed by Tukey’s post hoc test for multiple pairwise comparisons. A *p*-value of less than 0.05 was considered statistically significant.

For formulation optimization, the experimental data obtained from the FCCD were analyzed using multiple regression analysis to fit a second-order polynomial model [62]. The statistical significance of the model and individual model terms was evaluated by ANOVA. Model adequacy was assessed based on parameters such as the coefficient of determination (*R*^2^), adjusted *R*^2^, predicted *R*^2^, and lack-of-fit, where applicable. The optimized formulation was selected using the desirability function in Minitab^®^ [63]. All statistical analyses and DoE-based optimization were performed using Minitab version 22.0 (Minitab Inc., State College, PA, USA).

## 3. Results and Discussion

### 3.1. Preformulation and Absorption Enhancer Screening

Prior to QbD-based formulation optimization, preformulation studies and absorption enhancer screening were performed to identify the key formulation constraints for oral tirzepatide delivery [64]. This section summarizes the physicochemical properties of tirzepatide, its compatibility with candidate excipients, the gastric acid-neutralizing capacity of C10, and the absorption-enhancing potential of C10 in comparison with SNAC [23]. Collectively, these evaluations provided the scientific basis for selecting C10 as the absorption enhancer and for establishing the formulation strategy used in the subsequent development stage.

#### 3.1.1. Morphology

**Figure 1:**
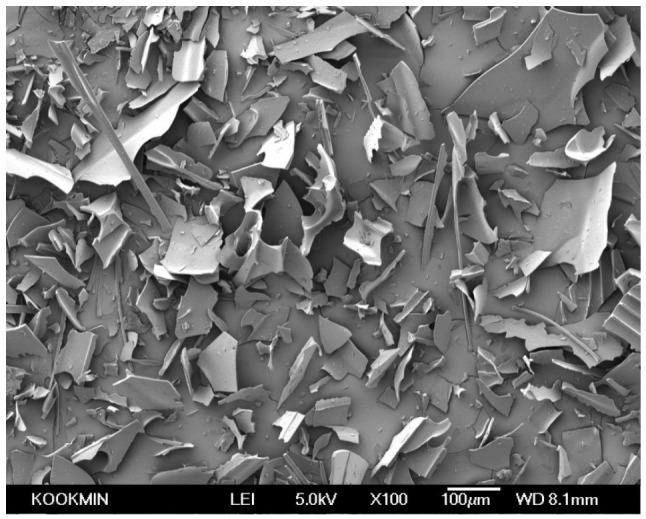
Scanning electron microscopy (SEM) micrographs of tirzepatide at 100× magnification, showing the irregular plate-like morphology and varied particle size distribution.

The particle morphology of tirzepatide was examined using SEM. As shown in Figure 1, the API particles exhibited predominantly irregular, angular, plate-like structures with relatively smooth and planar surfaces. Smaller fragments and fine particles were also observed among the larger particles, indicating a heterogeneous particle population. Image-based analysis of 100 randomly selected particles from representative SEM micrographs using ImageJ software yielded a mean particle size of 103.4 µm. These findings suggest that the raw material consists of coarse, plate-like particles accompanied by smaller particulates, which may influence powder flowability and blend uniformity during downstream tablet manufacturing.

#### 3.1.2. Solubility Profiling of Tirzepatide

As summarized in Table 4, tirzepatide exhibited solvent- and pH-dependent solubility. It was freely soluble in methanol (114.56 mg/mL) and distilled water (120.99 mg/mL), with rapid equilibration, whereas it was practically insoluble in ethanol and acetonitrile. As a 39-amino-acid peptide, tirzepatide contains multiple ionizable functional groups and therefore does not possess a single ionization constant (pKa). Instead, its pH-dependent solubility is governed by the combined ionization behavior of these groups and is closely related to its isoelectric point (pI).

More importantly, tirzepatide showed a highly pH-dependent solubility profile, which is consistent with its ionizable peptide structure. The API was below the quantification limit and practically insoluble under strongly acidic conditions (pH 1.2–4.0). After marginal detection at pH 5.0 (0.01 mg/mL). This practically undetectable solubility observed in the acidic range (pH 1.2–5.0) is consistent with the peptide approaching its isoelectric point, where its net charge becomes minimal, thereby promoting aggregation and precipitation. Subsequently, its aqueous solubility increased markedly under near-neutral to alkaline conditions, reaching 158.98 mg/mL at pH 8.0. These findings provide an important rationale for the oral formulation strategy used in this study. Because the formulation was designed to promote gastric absorption, the strongly acidic environment of gastric fluid could substantially limit tirzepatide dissolution and subsequent absorption. Therefore, modulation of the local microenvironmental pH toward near-neutral conditions, approximately above pH 6.0, is considered essential to promote rapid solubilization of tirzepatide in the stomach [15].

#### 3.1.3. Physicochemical Compatibility of Excipients

A physicochemical compatibility study was conducted to screen suitable excipients for the prototype formulation. Binary mixtures of tirzepatide and 13 candidate excipients (1:1, *w*/*w*) were evaluated after 4 weeks of storage under refrigerated (4 °C) and ambient (25 °C/60% RH) conditions [65]. As summarized in Table 5, macroscopic observation confirmed that all mixtures maintained their original white powder state without signs of physical instability, such as discoloration, caking, or liquefaction. Subsequent HPLC analysis revealed that all evaluated binary mixtures yielded drug recovery rates between 90% and 110% across all storage conditions. These findings indicate robust physicochemical compatibility between tirzepatide and the candidate excipients, confirming their suitability for inclusion in the final formulation design [35].

#### 3.1.4. Gastric Fluid Neutralization Capacity of Sodium Caprate (C10)

Modulation of the local gastric pH is an important strategy in absorption enhancer-based oral peptide delivery, as exemplified by the SNAC-containing oral semaglutide formulation. In this study, 0.01 N HCl was used as a simplified acidic challenge medium to evaluate the acid-neutralizing capacity and dispersion behavior of C10. This medium was not intended to fully reproduce the native composition of gastric fluid; rather, it was designed to reflect the weakly buffered nature of fasted gastric contents. In the human model, dilution of 50 mL of 0.01 N HCl with 120 mL of purified water resulted in a final acid load of approximately 2.94 mmol/L, which is in the same order as the low buffer capacity reported for fasted human upper gastrointestinal fluids. In the beagle model, dilution of 25 mL of 0.01 N HCl with 10 mL of purified water resulted in a final acid load of approximately 7.14 mmol/L. Because no additional buffer salts were included, this model maintained a low-ionic-strength acidic environment. The initial pH of the acidic medium was 2.09, closely matching the reported average fasted gastric pH of conscious beagle dogs, approximately pH 2.05. Thus, the volume-adjusted models were considered suitable as simplified low-buffer acidic systems for comparing the pH-shifting effect of C10 under beagle and human dosing conditions [66].

As presented in Table 6, C10 showed a dose-dependent acid-neutralizing effect. In the beagle model, even 100 mg of C10 increased the medium pH to a near-neutral range (pH 6.14–7.60), whereas the human model showed a clearer dose dependency. In the human model, 100 mg of C10 increased the pH only to 4.98 after 45 min, which remained below the pH range favorable for tirzepatide dissolution. In contrast, 300 and 500 mg of C10 increased the pH above 6.0, reaching pH 6.19–6.47 and providing a more favorable microenvironment for tirzepatide solubilization.

These findings are consistent with the pH-dependent solubility profile of tirzepatide observed in the preceding study. Because tirzepatide was practically insoluble under strongly acidic conditions but showed markedly improved solubility near neutral pH, the pH-shifting effect of C10 is directly relevant to the initial dissolution step of the oral formulation. Considering the larger total fluid volume in the human model and the insufficient pH elevation observed with 100 mg of C10, 300 mg was selected as the minimum C10 level for subsequent formulation development.

#### 3.1.5. Permeability Enhancement in Caco-2 Cell Monolayers

The epithelial permeability of tirzepatide and the effects of candidate absorption enhancers were evaluated using Caco-2 cell monolayers. Prior to transport studies, monolayer integrity was confirmed by TEER values exceeding 300 Ω·cm^2^ [30]. Preliminary cytotoxicity was assessed using MTT and LDH assays to determine appropriate enhancer concentrations (Figure S1). Cell viability was generally maintained above 80%; however, C10 at 10 mM showed a slight reduction in viability. The corresponding LDH results did not indicate severe membrane disruption, suggesting that the observed effect was limited under the tested conditions. Based on these results, SNAC (10 and 20 mM) and C10 (2.5, 5, and 10 mM) were selected for co-administration with tirzepatide (200 μM) to evaluate the balance between permeability enhancement and cellular integrity.

**Figure 2:**
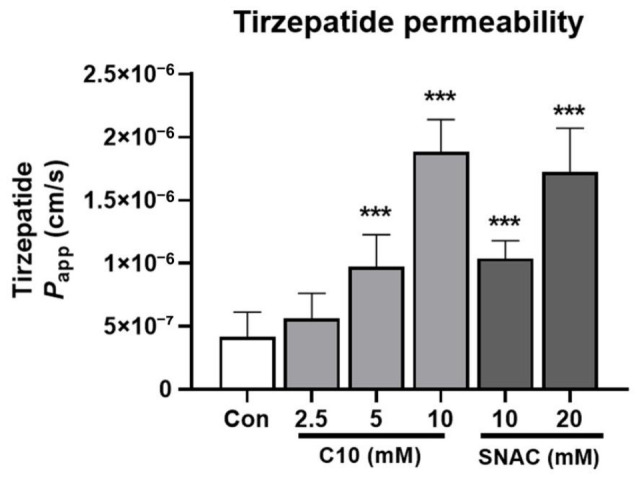
Effects of C10 and SNAC on the apparent permeability of tirzepatide across Caco-2 cell monolayers. The apparent permeability coefficient (*P*_app_) of tirzepatide was determined in the absence or presence of C10 and SNAC at the indicated concentrations. Data are expressed as mean ± SD (*n* = 3). *** *p* < 0.001 versus tirzepatide alone.

The apparent permeability coefficient (*P*_app_) of tirzepatide alone was inherently low ($4.49\times 10^{-7}cm/s$), reflecting the limited epithelial transport of the macromolecular peptide. Co-administration with absorption enhancers increased *P*_app_ in a concentration-dependent manner across the treatment groups (Figure 2, Table S1). Among the tested conditions, 10 mM C10 produced the highest *P*_app_ value ($1.84\times 10^{-6}cm/s$), corresponding to approximately a 4.1-fold increase over the control (*p* < 0.001). In comparison, 20 mM SNAC increased *P*_app_ to $1.53\times 10^{-6}cm/s$, corresponding to a 3.4-fold increase (*p* < 0.001). Although C10 was applied at half the molar concentration of SNAC, it showed a higher permeability-enhancing effect under the tested conditions, with an approximately 1.2-fold higher *P*_app_ than 20 mM SNAC. These findings support the selection of C10 as the absorption enhancer for subsequent oral tirzepatide formulation development.

#### 3.1.6. Preliminary In Vivo Screening in Rats

**Figure 3:**
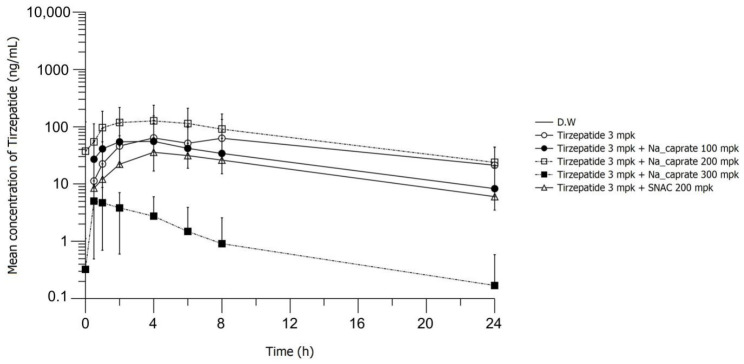
Mean plasma concentration–time profiles of tirzepatide in rats after oral co-administration with C10 or SNAC.

Quantitative analysis of rat plasma samples by UHPLC-MS/MS showed good calibration linearity (*r* > 0.99). The PK parameters and plasma concentration–time profiles are summarized in Table 7 and Figure 3, respectively. As expected, tirzepatide was not detected in the negative control group (G1, DW). The API-alone group (G2) showed measurable systemic exposure, with a *C*_max_ of 75.8 ng/mL and an area under the plasma concentration–time curve from time zero to the last quantifiable concentration (*AUC*_last_) of 1480 h·ng/mL.

Co-administration with C10 produced a non-linear exposure response. At 100 mg/kg (G3), C10 did not improve systemic exposure compared with the API-alone group. In contrast, increasing the C10 dose to 200 mg/kg (G4) resulted in the highest exposure among all groups, with a *C*_max_ of 157 ng/mL and an *AUC*_last_ of 2070 h·ng/mL. This result suggests that 200 mg/kg C10 provided the most favorable absorption-enhancing condition in this preliminary rat screening. However, further increasing the C10 dose to 300 mg/kg (G5) markedly reduced systemic exposure, with a *C*_max_ of 5.25 ng/mL and an *AUC*_last_ of 26.1 h·ng/mL. This indicates that the absorption-enhancing effect of C10 was not simply dose proportional and may be compromised at excessive levels, possibly due to unfavorable effects on drug dispersion, solubilization, or epithelial transport.

The SNAC-treated group (G6, 200 mg/kg) showed lower systemic exposure than both the API-alone group and the C10 200 mg/kg group, with a *C*_max_ of 36.1 ng/mL and an *AUC*_last_ of 852 h·ng/mL. Therefore, under the present experimental conditions, SNAC did not provide a clear absorption advantage over tirzepatide alone. Overall, these preliminary rat PK results indicate that the absorption-enhancing effect was strongly dependent on enhancer type and dose. Among the tested conditions, C10 at 200 mg/kg produced the highest systemic exposure, suggesting that C10 has an optimal effective range for enhancing tirzepatide absorption and that increasing the enhancer dose does not necessarily lead to improved systemic exposure.

### 3.2. QbD-Based Formulation and Process Optimization

Following the selection of C10 as the absorption enhancer, a QbD-based strategy was applied to optimize both formulation composition and manufacturing process conditions for the immediate-release tirzepatide tablet. First, the QTPP and CQAs were defined to establish the target product performance, with particular emphasis on rapid dissolution, acceptable mechanical integrity, and manufacturability [42]. Risk assessment using PHA and FMEA was then performed to identify high-risk formulation and process variables affecting these CQAs [31]. Based on this assessment, binder/disintegrant ratios were selected as key CMAs related to dissolution behavior, while hammer milling parameters, including rotation speed and sieve size, were selected as CPPs affecting granule flowability and tablet mechanical strength. Sequential FCCD-based optimization was subsequently conducted to define the formulation and process design spaces. This section describes how the identified CMAs and CPPs were optimized to achieve the target dissolution profile while maintaining acceptable tablet quality and process robustness.

#### 3.2.1. Establishment of QTPP, CQAs, and Risk Assessment

To systematically control the quality risks associated with the formulation and manufacturing of the immediate-release tirzepatide tablet, a QbD framework was implemented [67]. The development process began with the definition of the QTPP, followed by the identification of CQAs that were directly linked to the intended product performance. Quality risk management (QRM) tools, including PHA and FMEA, were then applied to assess the potential impact of formulation and process variables on the predefined CQAs [68].

For the PHA, risk levels were categorized as low (minimal impact, effectively controlled by standard operating procedures), medium (moderate impact requiring specific control strategies), and high (significant impact necessitating comprehensive optimization) (Table 8A). Subsequently, FMEA was conducted to provide a more detailed, quantitative assessment. The RPN was calculated by multiplying the scores of Severity (S), Occurrence (O), and Detectability (D), each rated on a scale of 1 to 5. Variables with an RPN exceeding a predefined threshold of 30 were classified as high-risk.

Rapid dissolution was considered a critical requirement for the oral delivery of tirzepatide, as it can support drug availability at the absorption site and reduce the risk of insufficient dissolution under gastrointestinal conditions. Accordingly, the type and proportion of binders and disintegrants were identified as CMAs specifically due to their opposing mechanistic impacts on the dissolution profile. Binders establish interparticulate solid bridges that inherently reduce matrix porosity and retard solvent ingress, thereby delaying drug release. Conversely, disintegrants rely on rapid capillary wicking and swelling-induced volume expansion to generate internal disruptive stresses that overcome these cohesive bonds, triggering rapid matrix fragmentation. Optimizing this delicate balance is essential solely to ensure the rapid and synchronized dissolution of both the peptide and the permeation enhancer. In parallel, the hammer milling process was evaluated as a critical process step (CPP) because sieve size and rotation speed dictate the particle size distribution (PSD) and micromeritic properties of the granules. While PSD theoretically influences dissolution kinetics by altering the available surface area, its precise control is predominantly critical for maintaining the mechanical integrity and flowability of this highly C10-loaded formulation (~75% *w*/*w*). Due to the weak interparticulate bonding and poor compactability inherent to C10-rich granules, aggressive milling conditions are prone to generating an excessive fraction of fines. Mechanistically, these fine particles fill the intergranular voids and diminish the air permeability of the powder bed, thereby sharply increasing the susceptibility to air entrapment during compaction. Upon decompression, the expansion of entrapped air within this weakened matrix structure can trigger severe physical defects such as lamination or excessive friability. To evaluate and mitigate the risk of these tableting failures, tablet friability (representing mechanical integrity) and Carr’s Index (representing flowability) were designated as key response variables for the subsequent process optimization.

Based on the FMEA results, variables with a RPN exceeding 30 were selected for further evaluation (Table 8B). The identified high-risk formulation and process variables, including binder/disintegrant ratios and hammer milling parameters, were optimized using FCCD to model their main and interaction effects on the predefined responses. This FCCD-based optimization enabled the establishment of a formulation and process strategy for achieving consistent dissolution performance and acceptable tablet quality [24].

#### 3.2.2. Formulation Optimization (CMAs)

To screen binder and disintegrant candidates for subsequent formulation optimization, a three-level, two-factor general factorial design was performed. Binder type and disintegrant type were evaluated as formulation-related material attributes, and their main effects on the dissolution *f*_2_ were analyzed by ANOVA, as summarized in Table 9A. The results showed that binder type significantly affected *f*_2_ at pH 1.2 (*p* < 0.05), whereas disintegrant type was the significant factor at pH 4.0. At pH 6.8, both binder and disintegrant types significantly influenced the dissolution similarity factor. Tukey’s post hoc analysis was then conducted to compare individual excipient levels and identify the binder and disintegrant candidates to be used in the subsequent FCCD optimization (Table 9B).

Because the formulation was designed to promote rapid drug release under gastric conditions, particular emphasis was placed on the pH 1.2 dissolution data during excipient selection. K25 was excluded because it produced an initial burst release and failed to satisfy the *f*_2_ similarity criterion, being classified into group “b”. In contrast, VA64 was consistently assigned to group “a” across all tested media, indicating the closest similarity to the target dissolution profile. To achieve more robust dissolution behavior, a dual-binder strategy was adopted: VA64 was selected for its overall balanced performance, while K90 was retained because it provided favorable initial dissolution at pH 1.2.

For disintegrants, croscarmellose sodium was excluded because it failed to meet the similarity criterion at pH 4.0 and pH 6.8, resulting in group “b” classification. SSG was consistently assigned to group “a” under all dissolution conditions, indicating dissolution behavior similarly to the target dissolution profile. CROS was also retained as a candidate because, although it did not show broad similarity across all pH conditions, it was classified into group “a” at the critical pH 1.2 condition, suggesting its potential contribution to early-stage drug release. Consequently, the selected binder pair, K90/VA64, and disintegrant pair, SSG/CROS, were used to define the formulation factors for subsequent FCCD optimization. The FCCD was then applied to quantitatively model the interaction effects of these paired excipients and optimize their mixing ratios.

Preliminary screening showed that single-binder or single-disintegrant systems were insufficient to match the target dissolution profile across all tested media. Although formulations containing individual excipients met the similarity criterion (*f*_2_ ≥ 50) in two of the three media, they showed either excessively rapid or delayed release in the remaining medium. Therefore, a binary excipient strategy was adopted. While keeping the total amounts of the binder and disintegrant fractions constant, the internal mixing ratios of the selected excipients were varied as independent formulation factors. To systematically evaluate the combined and interaction effects of these excipients and establish a robust formulation within the QbD framework, a two-factor FCCD was performed.

For the FCCD matrix, the total binder amount was fixed at 20 mg. The amount of K90 was assigned as *X*_1_ and varied from 0 to 20 mg, while the amount of VA64 was adjusted accordingly. Similarly, the total disintegrant amount was fixed at 32 mg, and the amount of SSG was assigned as *X*_2_ and varied from 0 to 32 mg, with the remaining portion composed of CROS. This design generated 13 experimental runs, including five center points. A total of 14 response variables (*Y*_1_–*Y*_14_), comprising the *f*_2_ values and dissolution percentages at the time points used for *f*_2_ calculation across pH 1.2, 4.0, and 6.8 media, were quantified to evaluate the main and interaction effects of the formulation factors (Table S2). Specifically, *Y*_1_–*Y*_5_ corresponded to the dissolution percentages at the selected time points in pH 1.2 medium, while *Y*_6_ represented the *f*_2_ value for pH 1.2. Similarly, *Y*_7_–*Y*_9_ and *Y*_11_–*Y*_13_ corresponded to the dissolution percentages at the selected time points in pH 4.0 and pH 6.8 media, respectively, whereas *Y*_10_ and *Y*_14_ represented the corresponding *f*_2_ values for pH 4.0 and pH 6.8. These response variables were used to assess both time-dependent dissolution behavior and overall similarity to the target dissolution profile.

Experimental data were analyzed by RSM using Minitab software. The relationships between the formulation factors and response variables were fitted to second-order polynomial models (Table 10A and Table S3), and model significance and adequacy were assessed by ANOVA (Table 10B and Table S4). Among the 14 response models, *Y*_5_ and *Y*_9_ were not statistically significant (*p* > 0.05) and showed no meaningful predictive ability (predicted *R*^2^ = 0.00%). Therefore, these responses were excluded from the multiple-response optimization (MRO) to avoid introducing unreliable constraints into the design space.

The remaining 12 response models were statistically significant (*p* < 0.05). Although some models showed relatively low coefficients of determination (*R*^2^ = 48.58–66.55%), they were retained because the purpose of this RSM analysis was to identify the overall effects and interaction trends of the formulation factors rather than to precisely predict each individual response value. In addition, variability in early-stage dissolution responses is expected during formulation screening because drug release is affected by multiple formulation and process-related factors. As the main effects and interaction patterns of the key formulation variables were consistently observed across most responses, the models were considered suitable for defining the optimization space and guiding selection of the final composition.

**Figure 4:**
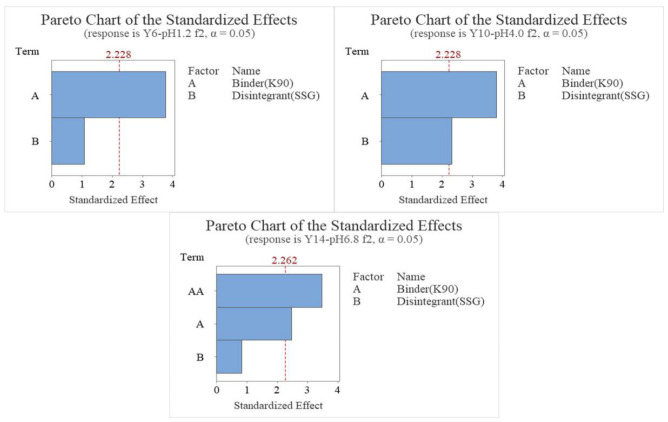
Pareto charts of representative *f*_2_ responses for CMA optimization. AA represents the interaction effect in the Design of Experiments (DoE).

**Figure 5:**
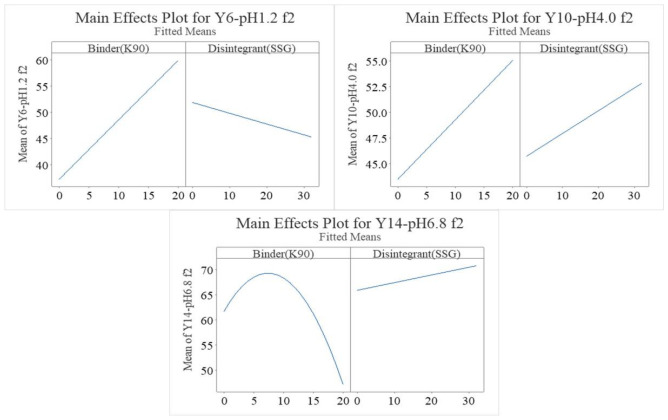
Main effects plots of binder and disintegrant ratios on representative *f*_2_ responses.

To maintain the hierarchy of the polynomial regression models, all first-order terms (*X*_1_ and *X*_2_) were retained regardless of their individual *p*-values. Backward elimination was then applied to simplify the models by removing non-significant interaction and quadratic terms (*p* ≥ 0.05), while preserving model hierarchy [69]. Based on the refined models, Pareto charts (Figure 4 and Figure S2) and main effects plots (Figure 5 and Figure S3) were generated to visualize the relative influence of the binder (*X*_1_) and disintegrant (*X*_2_) ratios on the selected responses.

**Figure 6:**
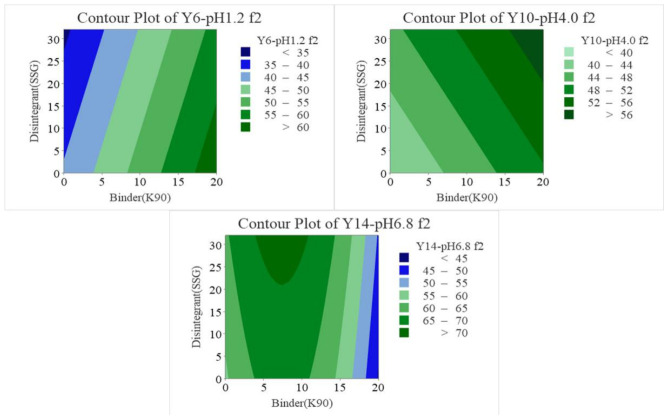
Contour plots of representative *f*_2_ responses for binder/disintegrant ratio optimization.

Pareto analysis of the representative *f*_2_ responses showed that the binder ratio (*X*_1_, K90) was the dominant formulation factor affecting dissolution similarity. For the pH 1.2 response (*Y*_6_), only *X*_1_ exceeded the statistical significance threshold. For the pH 6.8 response (*Y*_14_), both the linear and quadratic terms of *X*_1_ were significant, suggesting that the binder ratio may influence dissolution similarity in a non-linear manner. In contrast, the effect of the disintegrant ratio (*X*_2_, SSG) was statistically significant only in the pH 4.0 response (*Y*_10_), where its effect remained smaller than that of *X*_1_. These results indicate that the binder ratio primarily governed the representative *f*_2_ responses, while the disintegrant ratio contributed more selectively under the pH 4.0 condition. Therefore, multi-response optimization was required to balance the dominant binder effect with the medium-specific contribution of the disintegrant ratio and to achieve the target dissolution profile across all tested media. To visualize the combined effects of the binder (*X*_1_) and disintegrant (*X*_2_) ratios, response surface data for the representative *f*_2_ responses were presented as two-dimensional contour plots (Figure 6 and Figure S4).

**Figure 7:**
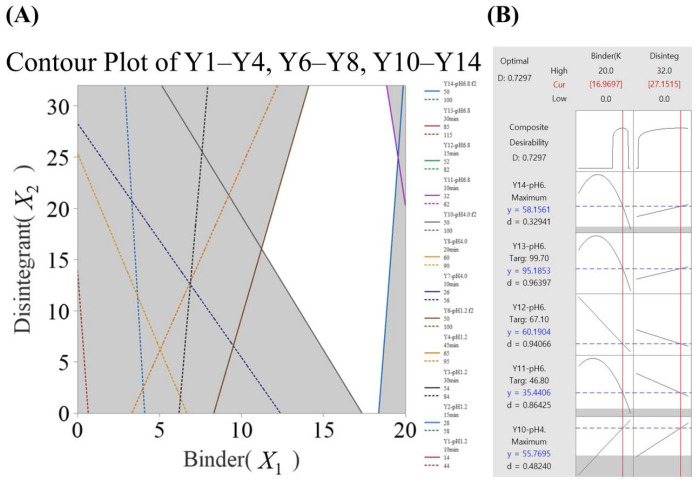
Design space and desirability-based optimization of binder/disintegrant ratios. (**A**) Overlaid contour plot for multiple dissolution responses. Overlaid contour plot for multiple dissolution responses. The white region represents the Design Space (DS) where all criteria are met, while the grey areas indicate regions that fail to satisfy the acceptable criteria. The solid and dashed lines indicate the boundaries of the acceptable ranges for each specific response. (**B**) Desirability plot showing the predicted optimal point. The vertical red lines represent the optimized settings for the formulation variables.

Subsequently, a MRO strategy was applied to identify an optimized formulation satisfying the predefined dissolution criteria. For the time-point dissolution responses, the values of the target dissolution profile were used as target values, whereas the *f*_2_ responses were set to be maximized with a lower limit of 50. By superimposing these criteria, an overlaid contour plot was constructed to define the design space (Figure 7A) [70]. The unshaded region represents the formulation range in which the predefined dissolution criteria were simultaneously satisfied.

To identify the optimal formulation within the established design space, MRO was performed using the desirability function. The optimization criteria were consistent with those used to construct the overlaid contour plot; however, different weights were assigned according to the relative importance of each response [71]. The time-point dissolution responses were assigned a lower weight of 0.1, whereas the *f*_2_, which represents the overall similarity to the target dissolution profile, was assigned a higher weight of 1.0 and set to be maximized. This weighting strategy was applied because simultaneous strict matching of all time-point dissolution values can overly restrict the optimization space in a multi-response system. Therefore, minor deviations in individual dissolution values were allowed within acceptable limits, while greater priority was placed on maximizing the overall *f*_2_ profile.

Under these conditions, the optimization generated a composite desirability of 0.7297 (Figure 7B). The predicted optimal factor settings were 16.9697 mg for K90 (*X*_1_) and 27.1515 mg for SSG (*X*_2_). For practical manufacturing and weighing feasibility, these values were rounded to the nearest integer [72]. Considering the fixed total amounts of binder and disintegrant fractions, the optimized formulation was set at 17 mg K90 and 3 mg VA64 for the binder phase, and 27 mg SSG and 5 mg CROS for the disintegrant phase.

#### 3.2.3. Process Optimization (CPPs)

Following formulation optimization, CPPs were evaluated to improve tablet mechanical integrity and process robustness during scale-up-oriented manufacturing. Because the absorption enhancer C10 accounted for approximately 75% of the total formulation weight, the mechanical behavior of the powder blend was largely governed by the properties of C10. Although preliminary slugging did not markedly affect the predefined CQAs, subsequent hammer milling produced a heterogeneous particle size distribution with an excessive fraction of fines (Table S5B). This tendency was likely associated with the weak interparticulate bonding and poor compactability of the C10-rich granules, which made them susceptible to fragmentation under milling stress [45]. The excessive generation of fines was considered a major contributor to tablet defects during compression. During die filling and compaction, fine particles can occupy interparticulate voids and reduce powder bed permeability, thereby increasing the likelihood of air entrapment. Upon decompression, expansion of the entrapped air, together with elastic recovery of the compact, may exceed the tensile strength of the C10-rich tablet matrix. This mechanism can promote lamination, which is characterized by transverse cracking or separation of the tablet.

To mitigate these mechanical defects and improve granule flowability, hammer mill rotation speed (*X*_3_) and sieve size (*X*_4_) were selected as primary CPPs based on the preceding risk assessment. In preliminary trials, milling speeds above 2000 rpm consistently induced lamination; therefore, the operating range of *X*_3_ was limited to 200–1000 rpm to reduce the risk of tableting failure. The sieve size (*X*_4_) was set within 1.0–3.0 mm according to the available equipment specifications. To quantitatively evaluate the effects of these milling parameters, tablet friability (*Y*_15_) and CI (*Y*_16_), representing tablet mechanical integrity and granule flowability, respectively, were designated as response variables [45].

A two-factor FCCD was used to define the process optimization space for the selected CPPs. The experimental matrix consisted of 13 randomized runs, including five center points (Table S5B). RSM was performed using Minitab software to fit second-order polynomial regression models linking the CPPs to the measured responses (Table 11A). The statistical significance and adequacy of the models were assessed by ANOVA (Table 11B) [73]. Both response models were statistically significant (*p* < 0.05), with coefficients of determination (*R*^2^) ranging from 82.93% to 93.64%. The model fit statistics indicated that the models adequately described the effects of the process variables on tablet friability and granule flowability. Therefore, these models were considered suitable for defining the process design space.

**Figure 8:**
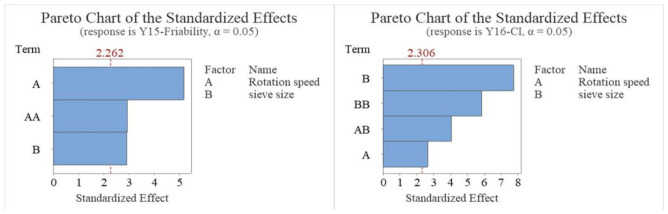
Pareto charts of CPP effects on tablet friability and granule flowability. AA and BB represent the quadratic effects of the respective factors, while AB represents the interaction effect between factors A and B.

**Figure 9:**
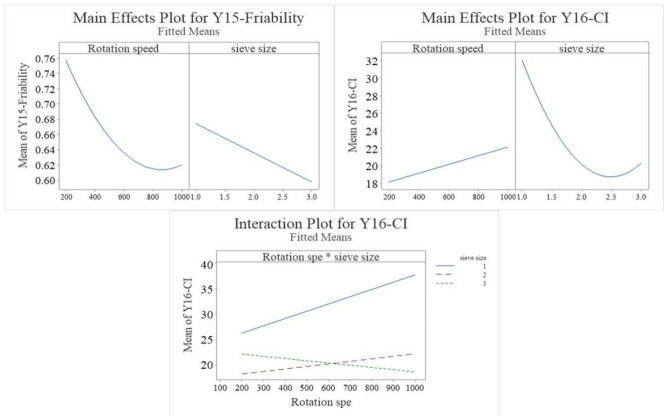
Main effects and interaction plots of hammer milling parameters on tablet friability and Carr’s index (CI). The asterisk (*) denotes the interaction effect between the two factors.

The standardized effects of the CPPs on the evaluated responses were analyzed using Pareto charts (Figure 8), main effects plots, and interaction plots (Figure 9). For tablet friability (*Y*_15_), Pareto analysis identified rotation speed (*X*_3_) as the dominant process variable. Milling speed can affect particle size distribution, surface characteristics, and granule morphology, which in turn influence powder packing, air entrapment, and compact mechanical integrity. In this study, friability showed a non-linear dependence on rotation speed. Acceptable mechanical integrity was maintained at rotation speeds of ≥600 rpm, whereas markedly increased friability was observed at 200 rpm. This suggests that excessively low milling speed may provide insufficient milling energy to form granules with favorable compactability, making 200 rpm unsuitable for maintaining tablet robustness.

This behavior may also be associated with the high C10 loading (~75% *w*/*w*) in the formulation. As an amphiphilic fatty acid salt, C10 can exhibit lubricant-like behavior during powder processing. At low rotation speed, prolonged residence in the milling chamber may increase frictional contact between C10-rich granules and the milling screen. This may promote surface smearing or coating-like redistribution of C10 on granule surfaces, thereby reducing interparticulate bonding during subsequent compaction [74]. Therefore, rotation speeds of ≥600 rpm were considered more suitable within the evaluated range because they may reduce excessive residence time and help maintain tablet mechanical integrity.

**Figure 10:**
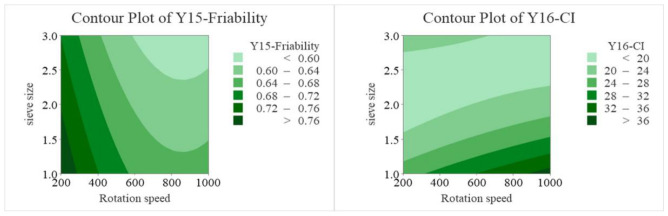
Contour plots of tablet friability and Carr’s index (CI) for hammer milling process optimization.

Regarding granule flowability, sieve size (*X*_4_) had the strongest effect on CI (*Y*_16_), along with significant quadratic and interaction effects (Figure 8). The interaction plot (Figure 9) showed that, when the smallest sieve size (1.0 mm) was used, CI increased at higher rotation speeds, indicating reduced flowability. In contrast, the larger sieve size (3.0 mm) maintained relatively low CI values across the tested speed range. These results suggest that sieve size is a key CPP for controlling granule flowability, particularly under higher milling speeds. To define the operating range for the milling parameters (*X*_3_ and *X*_4_), two-dimensional contour plots were generated for tablet friability and CI (Figure 10).

**Figure 11:**
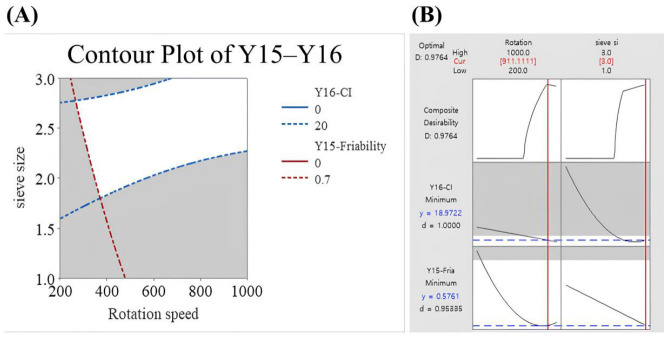
Design space and desirability-based optimization of hammer milling parameters. (**A**) Overlaid contour plot for tablet friability and Carr’s index (CI). The white region represents the Design Space (DS) where all criteria are met, while the grey areas indicate regions that fail to satisfy the acceptable criteria. The solid and dashed lines indicate the boundaries of the acceptable ranges for each specific response. (**B**) Desirability plot showing the predicted optimal process condition. The vertical red lines represent the optimized settings for the formulation variables.

Because the optimal condition for one response may not necessarily satisfy the other, MRO was performed to simultaneously meet the criteria for tablet mechanical integrity and granule flowability. The combined constraints were visualized using an overlaid contour plot (Figure 11A). The acceptance criteria were set based on pharmacopeial limits and practical considerations for process robustness. The friability limit was set at ≤0.7%, which is stricter than the conventional compendial limit of ≤1.0%, to provide an additional margin for potential batch-to-batch variability during scale-up. The CI criterion was set at ≤20, corresponding to fair or better flowability according to USP-based classification [75]. This criterion was selected to support consistent powder flow, die filling, and tablet weight uniformity during compression. By superimposing these criteria, the process design space was established. The unshaded region in the overlaid contour plot represents the operating range in which both friability and flowability criteria were simultaneously satisfied.

To identify the optimal process conditions within the established design space, MRO was performed. The objective function was set to minimize both tablet friability (*Y*_15_) and CI (*Y*_16_). Unlike the formulation optimization step, equal weights of 1.0 were assigned to both responses because tablet mechanical integrity and granule flowability were considered equally important for downstream tableting performance; inadequate flowability or poor mechanical strength represents a manufacturing failure that cannot offset one another [76].

Under these criteria, the model generated a composite desirability (D) of 0.9764. The predicted optimal settings were 911.11 rpm for rotation speed (*X*_3_) and 3.0 mm for sieve size (*X*_4_). For practical operation and equipment setpoint feasibility, the rotation speed was adjusted to 900 rpm [72]. Accordingly, 900 rpm and 3.0 mm were selected as the optimized hammer milling conditions for the dry granulation process.

### 3.3. Verification of the Optimized Formulation

Following the QbD-based optimization of CMAs and CPPs, the optimized formulation was experimentally verified to confirm whether the predicted formulation and process conditions could achieve the intended product performance [77]. The verification focused on two aspects: dissolution performance relative to the benchmark oral semaglutide tablet and the physical properties of the optimized granules and tablets [13]. Because the optimal C10 level for in vivo evaluation was not fully fixed at this stage, formulations containing 300 mg and 500 mg of C10 were both evaluated while maintaining the optimized binder/disintegrant composition. This verification step was intended to confirm that the optimized formulation could provide benchmark-comparable dissolution behavior while maintaining acceptable tablet mechanical integrity and granule flowability. To validate the statistical robustness of the predictive models, the prediction errors between the anticipated and observed values were evaluated. For dissolution performance, the observed *f*_2_ across all tested pH media (75 at pH 1.2, 58 at pH 4.0, and 68 at pH 6.8) consistently exceeded the model-predicted values (54, 55, and 58, respectively). Because a higher *f*_2_ value inherently indicates a stronger profile similarity to the benchmark drug, this positive deviation validates excellent formulation robustness beyond the model’s conservative estimations. Regarding the physical properties of the formulation, tablet friability showed a remarkably low prediction error of 5.45%. Although the CI exhibited a larger prediction error (26.32%), this was expected due to the inherently high variation in powder flow measurements at a laboratory scale; nevertheless, the observed CI successfully satisfied the acceptable compendial criteria. Detailed analytical results for these verification studies are discussed in Section 3.3.1 and Section 3.3.2.

#### 3.3.1. Comparative In Vitro Dissolution Profiles

**Figure 12:**
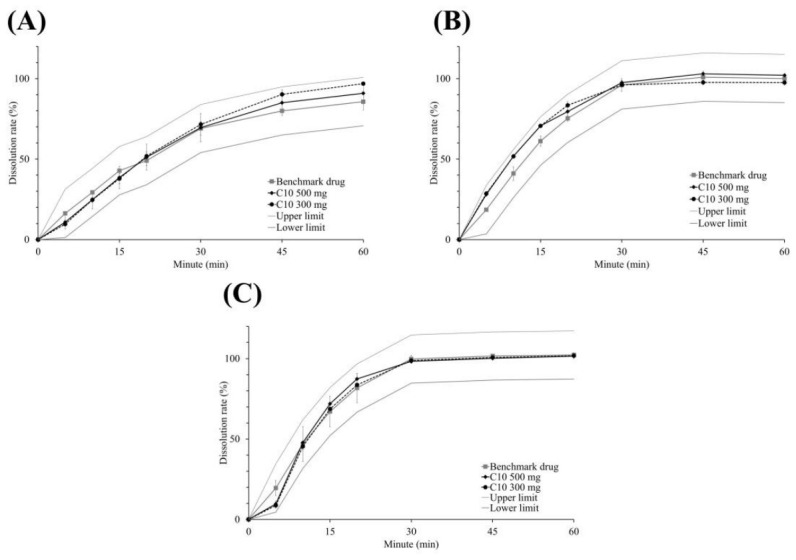
Dissolution profiles of optimized tirzepatide formulations compared with the benchmark drug across different dissolution media: (**A**) pH 1.2 containing 0.75% Brij 35; (**B**) pH 4.0 containing 0.75% Brij 35; and (**C**) pH 6.8 without surfactant. The optimized formulations containing 300 mg and 500 mg of sodium caprate (C10) were evaluated. The upper and lower solid lines represent the predefined acceptable range based on the benchmark drug.

The in vitro dissolution performance of the optimized formulations was evaluated against the benchmark drug, Rybelsus, across three dissolution media: pH 1.2 containing 0.75% Brij 35, pH 4.0 containing 0.75% Brij 35, and pH 6.8 without surfactant (Figure 12). Because the optimal C10 level for subsequent in vivo evaluation had not been fully determined at this stage, two formulations were prepared: the optimized formulation containing 500 mg of C10 and an additional formulation in which only the C10 level was reduced to 300 mg while maintaining the optimized binder/disintegrant composition.

Both formulations showed dissolution profiles comparable to that of the benchmark drug. The C10 500 mg formulation achieved *f*_2_ values of 68.22, 58.27, and 75.05 at pH 1.2, 4.0, and 6.8, respectively. Similarly, the C10 300 mg formulation showed *f*_2_ values of 56.0, 55.4, and 88.4 in the same media. Thus, both C10 levels satisfied the similarity criterion (*f*_2_ ≥ 50) across all tested dissolution conditions [78]. These results indicate that reducing the C10 level from 500 mg to 300 mg did not markedly alter the overall dissolution similarity once the binder/disintegrant ratios had been optimized.

Although Rybelsus contains a different API, it was used as a benchmark drug because it is currently the only marketed oral GLP-1 receptor agonist employing an absorption enhancer for fasting gastric absorption [79]. Mechanistically, interpreting these dissolution profiles in the context of gastrointestinal transit physiology and peptide stability further validates our formulation strategy. Oral GLP-1 receptor agonists like tirzepatide are highly susceptible to acidic and enzymatic degradation in the gastrointestinal tract. Because the benchmark drug relies on rapid tablet disintegration and dissolution in the fasting stomach (pH 1.2) to create a highly concentrated localized absorption zone, mimicking its rapid release profile at pH 1.2 is critical. The high *f*_2_ values obtained at pH 1.2 confirm that the optimized formulation rapidly co-releases tirzepatide and C10. This rapid dissolution behavior is physiologically advantageous, as it is hypothesized to promote rapid gastric or proximal intestinal absorption that effectively outpaces peptide degradation. Thus, similarity to the Rybelsus dissolution profile was regarded as a practical formulation target rather than evidence of pharmaceutical equivalence. The optimized binder/disintegrant system achieved benchmark-comparable dissolution profiles for both C10 300 mg and 500 mg formulations, supporting their use in subsequent in vivo PK evaluation.

#### 3.3.2. Physical Properties of the Optimized Granules and Tablets

To evaluate the practical applicability of the DoE-derived optimum, granules and tablets were manufactured using the optimized formulation and process parameters. The observed tablet friability was 0.58%, which matched the predicted value and satisfied the compendial requirement of ≤1.0%. This result supports that the optimized hammer milling condition, particularly the use of a higher rotation speed, was effective in maintaining tablet mechanical integrity, likely by reducing excessive residence time and limiting surface lubrication of the C10-rich granules.

The measured CI was 24, which was higher than the predicted value of 19 and slightly exceeded the predefined optimization target of ≤20. This deviation may be attributed to small-scale processing variability, manual handling during granule collection, and electrostatic effects during flowability measurement. Nevertheless, a CI of 24 is classified as “passable” according to USP-based flowability criteria, suggesting that the optimized granules retained acceptable flowability for laboratory-scale tableting [49]. However, given the highly cohesive nature of the C10-loaded formulation (~75% *w*/*w*), the possibility of weight variations due to reduced flowability during large-scale manufacturing cannot be completely excluded. If such issues arise during high-speed scale-up, they can be effectively mitigated by a formulation approach, such as incorporating a minor amount of extra-granular glidant (e.g., colloidal silicon dioxide). Overall, the optimized CMA and CPP conditions produced tablets with satisfactory mechanical integrity and acceptable granule flowability, although further process refinement may be required to improve flowability robustness during scale-up [80].

### 3.4. Comparative In Vivo Pharmacokinetic (PK) in Beagle Dogs

**Figure 13:**
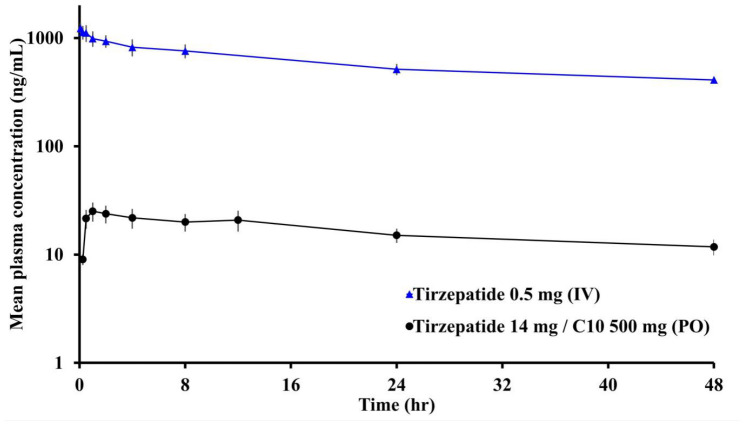
Plasma concentration–time profiles of tirzepatide in beagle dogs following intravenous (0.5 mg/animal) and oral administration of a C10-based tirzepatide tablet (14 mg tirzepatide and 500 mg C10 per animal). Data are presented as mean ± SD (*n* = 3).

To assess the oral absorption potential of the C10-based tirzepatide tablet, plasma concentration–time profiles were compared following intravenous administration of tirzepatide and oral administration of a tablet containing 500 mg of C10 in beagle dogs (Figure 13). Following intravenous administration of tirzepatide at 0.5 mg/animal, the *AUC*last was 28,257.78 ± 3140.75 h·ng/mL, and the half-life (T1/2) was 43.72 ± 7.43 h. The intravenous group served as the systemic exposure reference for evaluating the oral formulation. Following oral administration of the tirzepatide tablet containing 500 mg of C10, the Cmax was 25.17 ± 5.05 ng/mL, and the AUClast was 790.21 ± 137.45 h·ng/mL. Although systemic exposure following oral administration was limited relative to that following intravenous administration, measurable plasma concentrations of tirzepatide were observed, with a distinct concentration–time profile. These results demonstrate that oral absorption of tirzepatide can be achieved using a C10-based tablet formulation.

**Figure 14:**
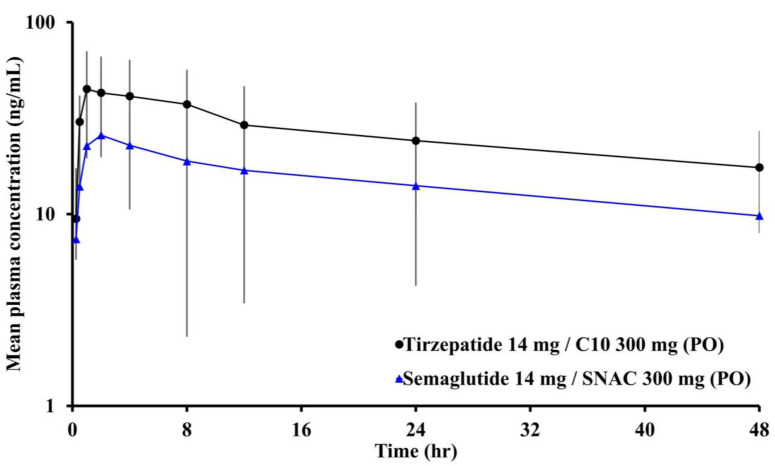
Comparative plasma concentration–time profiles of the optimized C10-based oral tirzepatide tablet and the SNAC-based oral peptide delivery benchmark, Rybelsus, in beagle dogs. The optimized formulation contained 14 mg tirzepatide and 300 mg C10 per animal. Data are presented as mean ± SD (*n* = 3).

Based on the initial PK evaluation and preliminary rat PK screening results, a second PK study was conducted to optimize the C10 content and assess the reproducibility of the C10-based oral delivery strategy (Figure 14 and Table 12). Following oral administration of the C10 300 mg formulation, the *C*_max_ was 46.49 ± 23.79 ng/mL, the *AUC*_last_ was 1261.03 ± 690.44 h·ng/mL, and the median *T*_max_ was 1.0 h. Compared with the C10 500 mg formulation, the C10 300 mg formulation achieved approximately 1.85-fold higher *C*_max_ and 1.60-fold higher *AUC*_last_.

The increase in systemic exposure despite reducing the C10 content from 500 to 300 mg indicates that the absorption-enhancing effect of C10 is not simply dose proportional. This paradoxical, dose-dependent absorption behavior has been similarly reported in the clinical development of Rybelsus, where co-formulating semaglutide with 300 mg of the absorption enhancer SNAC resulted in higher systemic exposure compared to a 600 mg SNAC dose [81]. Mechanistically, this paradoxical decrease in absorption at an excessively high enhancer concentration can be attributed to gastrointestinal fluid secretion. A 500 mg dose of C10 may create a severely hyperosmotic microenvironment or cause local mucosal irritation, triggering rapid water influx into the GI lumen. This physiological fluid secretion inevitably dilutes the highly concentrated, localized absorption zone of both the permeation enhancer and the peptide. Furthermore, an excessively high local salt concentration could induce a salting-out effect, potentially reducing the solubility of tirzepatide. Taken together with the preliminary rat PK screening results, these findings suggest that excessive amounts of C10 do not necessarily further enhance tirzepatide absorption and that an optimal C10 range may be required to maximize oral absorption.

The PK profile of the optimized C10 300 mg formulation was subsequently compared with that of Rybelsus, a commercially available SNAC-based oral peptide delivery benchmark. The Rybelsus group exhibited a *C*_max_ of 26.13 ± 37.56 ng/mL and an *AUC*_last_ of 712.03 ± 995.25 h·ng/mL, with considerable inter-subject variability. In comparison, the C10 300 mg formulation achieved approximately 1.78-fold higher mean *C*_max_ and 1.77-fold higher mean *AUC*_last_ than the Rybelsus benchmark group.

Because tirzepatide and semaglutide are distinct peptide therapeutics, these results should not be interpreted as evidence of direct bioequivalence or therapeutic superiority. Nevertheless, it is noteworthy that the C10-based tirzepatide formulation achieved a competitive mean systemic exposure relative to the commercially available SNAC-based oral peptide delivery benchmark. These findings support the potential of C10 as a promising strategy for next-generation oral peptide delivery.

## 4. Conclusions

In this study, a systematic QbD approach was applied to develop an oral immediate-release tablet formulation of tirzepatide, a dual GIP/GLP-1 receptor agonist. Preformulation and absorption enhancer screening demonstrated that C10 could contribute to both microenvironmental pH modulation and permeability enhancement, supporting its potential as an absorption enhancer for oral tirzepatide delivery. C10 was selected as the absorption enhancer based on its gastric acid-neutralizing capacity, permeability-enhancing effect in Caco-2 cell monolayers, and preliminary in vivo screening performance. Notably, the screening results suggested that the absorption-enhancing effect of C10 was not simply dose proportional, indicating the need to define an optimal effective range.

Within the QbD framework, formulation and process risks were systematically evaluated following the definition of QTPP and CQAs. Statistical screening and FCCD-based optimization successfully identified the optimal binder (K90/VA64) and disintegrant (SSG/CROS) ratios required to achieve dissolution profiles comparable to the benchmark oral semaglutide tablet across pH 1.2, 4.0, and 6.8 media. Process optimization was equally essential, as the high C10 content strongly influenced the mechanical behavior of the formulation. By evaluating hammer milling parameters—with a focus on particle surface roughness and shape frequency rather than solely the volume of fines—optimized conditions (900 rpm with a 3.0 mm sieve) were established. This process effectively reduced the risk of physical defects, ensuring robust granule flowability and acceptable tablet mechanical integrity (friability of 0.58%).

Both the 300 mg and 500 mg C10 formulations exhibited benchmark-comparable dissolution profiles (*f*_2_ > 50). However, in the beagle dog pharmacokinetic study, the 300 mg C10 formulation achieved higher systemic exposure (*C*_max_ of 46.49 ± 23.79 ng/mL, *AUC*_last_ of 1261.03 ± 690.44 h·ng/mL, and ~0.16% absolute bioavailability) compared to the 500 mg formulation. Similarly to the clinical precedent of oral semaglutide, this paradoxical decrease in absorption at a higher C10 dose suggests that excessive enhancer may create a severely hyperosmotic microenvironment, triggering rapid fluid secretion that dilutes the localized absorption zone. Therefore, 300 mg of C10 was determined to be the optimal enhancer level for balancing dissolution, permeation enhancement, and in vivo systemic exposure.

Overall, this study demonstrated that a QbD-based approach could be effectively applied to the development of a C10-based oral tirzepatide tablet by systematically optimizing both formulation composition and process conditions. The optimized C10 300 mg formulation achieved a dissolution profile comparable to the benchmark oral semaglutide tablet, acceptable tablet mechanical integrity, and measurable systemic exposure in beagle dogs. This study successfully proved the actual viability of oral tirzepatide administration; at the optimal C10 concentration (300 mg), the formulation effectively facilitated in vivo systemic entry, confirming successful systemic absorption. These findings support the feasibility of C10-mediated oral tirzepatide delivery and provide a rational basis for further formulation refinement and PK evaluation.

## Figures and Tables

**Table 1:** Summary of the Quality Target Product Profile (QTPP) and critical quality attributes (CQAs) for the oral tirzepatide tablet.

QTPP Elements	Target	Is This a CQA?	Justification
Route of administration	Oral	No	Targeted to enhance patient compliance and therapeutic convenience, effectively overcoming the limitations of conventional subcutaneous injections.
Dosage form & design	Immediate- Release Tablet containing Tirzepatide 14 mg	No	Designed to enable rapid disintegration and release of tirzepatide together with the absorption enhancer, thereby supporting local co-exposure at the absorption site.
Appearance	Intact oblong tablet	Yes	Important for patient acceptability, ease of swallowing, and visual detection of defects such as discoloration, chipping, or surface irregularities.
Mechanical Integrity (Hardness & Friability)	Hardness: 7–9 kP Friability: NMT 1.0%	Yes	High C10 loading may reduce compactability and interparticulate bonding, increasing the risk of lamination and friability. Because granule size distribution and tablet strength are influenced by milling and compression conditions, mechanical integrity was selected as a key CQA.
Assay	90.0% to 110.0% of label claim	Yes	Assay directly affects dose accuracy, therapeutic performance, and safety; therefore, it must be controlled within the predefined acceptance range.
Content uniformity	Conforms to USP <905> Uniformity of dosage units	Yes	Content uniformity ensures consistent distribution of tirzepatide among individual tablets, which is essential for dose uniformity and product consistency.
Impurity	Unknown impurities: NMT 0.2%, Total impurities: NMT 2.0%	Yes	Peptide APIs are susceptible to chemical degradation during manufacturing and storage. Therefore, impurity levels must be controlled to ensure product stability and patient safety throughout the intended shelf life.
Dissolution	Similar dissolution profile to the benchmark drug; *f*_2_ ≥ 50	Yes	Rapid and reproducible dissolution is required to promote co-release of tirzepatide and the absorption enhancer, supporting sufficient local co-exposure at the absorption site. Similarity to the benchmark drug dissolution profile was used as a formulation target for evaluating immediate-release performance.

Color coding indicates the initial risk level: green, non-CQA/low risk; yellow, CQA requiring routine control; red, high-risk CQA requiring strict control and optimization.

**Table 2:** Base composition and design of experiments (DoE) factors for the critical material attributes (CMAs) in the oral tirzepatide tablet formulation.

Ingredients	Amount (mg/tab)	DoE Factor
Tirzepatide	14.0	-
Lactose	97.3	-
Sodium caprate	500.0	-
Binder	20.0	Blending ratio (*X*_1_)
Disintegrant	32.0	Blending ratio (*X*_2_)
Magnesium stearate	6.7	-
Total weight	670.0	-

The total amounts of binder and disintegrant were fixed at 20.0 and 32.0 mg/tablet, respectively. *X*_1_ represents the amount of povidone K90 within the fixed binder fraction, with the amount of copovidone VA64 adjusted inversely. *X*_2_ represents the amount of sodium starch glycolate within the fixed disintegrant fraction, with the amount of crospovidone adjusted inversely.

**Table 3:** Operating ranges and design of experiments (DoE) factors for the critical process parameters (CPPs) in the sizing process.

Process Step	Process Parameter	Unit	Operating Range	DoE Factor
Sizing	Rotation speed	rpm	200–1000	*X*_3_
	Sieve size	mm	1.0–3.0	*X*_4_

**Table 4:** The results of solubility test of tirzepatide in various solvents (*n* = 3).

No.	Solvent	Solubility of Tirzepatide			
		Apparent Solubility	Equilibrium Solubility (mg/mL)		
			2 h	12 h	24 h
1	Water	++++++	120.99 ± 6.18	118.33 ± 6.14	120.58 ± 6.57
2	Ethanol	+	0.24 ± 0.10	0.24 ± 0.14	0.24 ± 0.14
3	Methanol	++++++	110.01 ± 5.62	113.31 ± 6.71	114.56 ± 6.73
4	Acetonitrile	+	N.D.	N.D.	N.D.
5	pH 1.2	+	N.D.	N.D.	N.D.
6	pH 2.0	+	N.D.	N.D.	N.D.
7	pH 3.0	+	N.D.	N.D.	N.D.
8	pH 4.0	+	N.D.	N.D.	N.D.
9	pH 5.0	+	N.D.	N.D.	0.01 ± 0.00
10	pH 6.0	+++++	53.20 ± 3.20	53.51 ± 3.23	53.67 ± 3.27
11	pH 6.8	+++++	53.08 ± 5.41	53.22 ± 5.52	53.74 ± 5.65
12	pH 7.0	+++++	66.64 ± 7.06	67.14 ± 10.22	67.50 ± 6.86
13	pH 8.0	++++++	156.57 ± 15.63	157.52 ± 16.16	158.98 ± 16.66
14	pH 9.0	++++++	103.50 ± 1.82	104.30 ± 2.49	104.94 ± 2.84
15	pH 10.0	++++++	121.86 ± 7.42	122.31 ± 8.14	122.96 ± 8.32
16	pH 11.0	+++++	54.35 ± 2.04	54.82 ± 2.34	55.08 ± 2.54
17	pH 12.0	++++	12.91 ± 1.05	12.86 ± 1.07	12.76 ± 1.07

+: Practically insoluble, ++++: Sparingly soluble, +++++: Soluble, ++++++: Easily soluble. N.D.: Not Detected.

**Table 5:** Drug–excipient compatibility of tirzepatide binary mixtures with various excipients (Assay %).

Category	Excipient	Ratio (*w*/*w*)	Refrigerated (4 °C)		Room Temperature (25 °C, 60% RH)	
			2 W	4 W	2 W	4 W
API	Tirzepatide	-	99.0	94.4	101.7	100.7
Filler	Microcrystalline cellulose	1:1	98.9	96.9	110.4	98.6
	Lactose monohydrate	1:1	99.2	101.0	103.1	91.5
	Mannitol	1:1	101.0	95.1	106.9	94.7
Disintegrant	Croscarmellose sodium	1:1	110.6	94.8	98.1	92.1
	Crospovidone	1:1	99.1	95.6	103.0	100.5
	Sodium starch glycolate	1:1	102.5	97.1	105.7	96.8
Lubricant	Magnesium stearate	1:1	109.6	108.8	108.7	109.6
	Talc	1:1	106.7	110.2	107.7	105.7
Binder	Povidone K90	1:1	101.4	98.2	99.6	103.8
	Povidone K25	1:1	100.5	99.6	101.3	98.9
	Copovidone VA64	1:1	110.4	110.2	110.3	111.6
Alkalizer	Sodium bicarbonate	1:1	101.6	104.9	100.8	91.4
Absorption enhancer	Sodium caprate	1:1	100.6	107.0	97.2	98.3
	Salcaprozate sodium	1:1	90.0	93.9	102.4	95.1

**Table 6:** pH changes after sodium caprate (C10) addition under simulated beagle and human gastric conditions.

Parameter	Simulated Beagle Gastric Condition			Simulated Human Gastric Condition		
	C10 100 mg	C10 300 mg	C10 500 mg	C10 100 mg	C10 300 mg	C10 500 mg
pH (15 min)	6.14	7.08	7.51	4.95	6.19	6.39
pH (45 min)	6.29	7.34	7.6	4.98	6.31	6.47

**Table 7:** Pharmacokinetic (PK) parameters of tirzepatide after oral co-administration with C10 or SNAC in rats.

Group	Dose	*T*_max_ (h)	*C*_max_ (ng/mL)	*AUC*_last_ (h⋅ng/mL)	*T*_1/2_ (h)
G1	Distilled water	0.0	0.0	NA	NA
G2	Tirzepatide 3 mg/kg (Control)	3.0 ± 2.4	75.8 ± 84.1	1480 ± 988	11.6 ± 5.7
G3	+C10 100 mg/kg	1.5 ± 1.5	58.5 ± 73.1	644 ± 860	5.9 ± 2.2
G4	+C10 200 mg/kg	2.8 ± 2.7	157 ± 102	2070 ± 1200	8.5 ± 0.1
G5	+C10 300 mg/kg	0.8 ± 0.6	5.25 ± 4.45	26.1 ± 37.4	11.9 ± 15.3
G6	+SNAC 200 mg/kg	1.5 ± 2.0	36.1 ± 78.8	852 ± 1300	7.1 ± 0.7

All groups except G1 received tirzepatide 3 mg/kg orally. Data are expressed as mean ± SD (*n* = 5–6). *T*_max_, time to reach maximum plasma concentration; *C*_max_, maximum plasma concentration; *AUC*_last_, area under the plasma concentration–time curve from time zero to the last measurable concentration; *T*_1/2_, terminal half-life. NA, not applicable.

**Table 8:** Risk assessment for the formulation and process variables: (**A**) Preliminary hazard analysis (PHA) and (**B**) Failure mode and effects analysis (FMEA).

(**A**)											
**Variables**	**Appearance**	**Assay**		**Uniformity**	**Impurities**			**Dissolution**		**Mechanical** **Integrity**	
Material Attributes											
Tirzepatide	Low	Low		Medium	Medium			Low		Low	
Filler	Low	Low		Low	Low			Medium		Medium	
Binder	Low	Low		Low	Medium			High		Low	
Disintegrant	Low	Low		Low	Low			High		Low	
Absorption enhancer	Low	Low		Low	Medium			Low		High	
Lubricant	Low	Low		Low	Low			Low		Medium	
Process Parameters											
Slugging	Low	Low		Low	Low			Low		Low	
Sizing	Low	Low		Low	Low			Medium		High	
Tableting	Low	Low		Low	Low			Medium		Medium	
(**B**)											
**Unit Operation** **/Material Attribute**	**Potential Failure Mode**		**Risk Details** **(Effect, Cause, and Strategy)**			***O***	***S***		***D***		**RPN**
API (Tirzepatide)	Segregation or peptide degradation		Effect: Content variation and impurity increase. Cause: Low API ratio and peptide instability. Strategy: Standardized blending, moisture/light control, IPC sampling.			3	2		2		12
Absorption enhancer	Insufficient or excessive enhancer level		Effect: Poor dissolution/absorption or reduced exposure at excessive levels. Cause: C10 affects pH modulation, permeability, and tablet properties. Strategy: Range setting based on neutralization, Caco-2, and preliminary rat PK screening.			3	3		2		18
Binder Ratio (*X*_1_)	Inappropriate binding strength; over- or under-binding		Effect: Dissolution failure and/or poor mechanical quality. Cause: Suboptimal binder ratio. Strategy: Dissolution screening and FCCD optimization.			3	5		3		45
Disintegrant Ratio (*X*_2_)	Insufficient swelling or wicking capacity		Effect: Delayed disintegration and dissolution failure. Cause: Suboptimal disintegrant ratio. Strategy: Dissolution screening and FCCD optimization.			3	4		3		36
Sizing (Hammer milling, *X*_3_ & *X*_4_)	Inappropriate particle size distribution (PSD)		Effect: Poor mechanical integrity, lamination, or high friability. Cause: Suboptimal rotation speed (*X*_3_) and sieve size (*X*_4_) Strategy: Sieve analysis, flowability/mechanical testing, and FCCD optimization.			4	4		2		32

Risk level color coding: Green indicates low risk, yellow indicates medium risk, and red indicates high risk. S, Severity; O, Occurrence; D, Detectability; RPN, Risk Priority Number (calculated as S × O × D).

**Table 9:** (**A**) Analysis of variance (ANOVA) and (**B**) Tukey’s post hoc test of dissolution similarity factors (*f*_2_) based on a full factorial design for various binders and disintegrants at pH 1.2, 4.0 (with 0.75% polyoxyethylene lauryl ether), and 6.8.

(**A**)							
**Factor**	**DF**	**pH 1.2**		**pH 4.0**		**pH 6.8**	
		***F*-Value**	***p*-Value**	***F*-Value**	***p*-Value**	***F*-Value**	***p*-Value**
Binder	2	12.27	0.020	4.76	0.088	15.89	0.013
Disintegrant	2	0.18	0.842	10.10	0.027	8.78	0.034
Error	4	-	-	-	-	-	-
*R*^2^ (%)		86.16		88.13		92.50	
(**B**)							
**Factor**	**Level**	**pH 1.2**		**pH 4.0**		**pH 6.8**	
		**Mean *f*_2_**	**Grouping**	**Mean *f*_2_**	**Grouping**	**Mean *f*_2_**	**Grouping**
Binder	Povidone K90	57.51 ± 5.08	a	61.17 ± 14.47	a	37.50 ± 7.07	b
	Povidone K25	32.36 ± 4.59	b	47.49 ± 14.96	a	51.11 ± 5.19	a
	Copovidone VA64	51.49 ± 6.71	a	60.76 ± 5.40	a	52.90 ± 8.21	a
Disintegrant	Croscarmellose Sodium	47.58 ± 16.86	a	43.99 ± 12.80	b	40.49 ± 9.87	b
	Sodium starch glycolate	45.35 ± 11.90	a	66.14 ± 8.96	a	52.90 ± 10.11	a
	Crospovidone	48.43 ± 13.28	a	59.30 ± 3.74	a, b	48.13 ± 6.35	a, b

DF: degrees of freedom; *R*^2^: coefficient of determination. Different letters (a, b) indicate statistically significant differences between groups based on Tukey’s post-hoc test (*p* < 0.05).

**Table 10:** FCCD regression models and ANOVA results for CMA optimization. (**A**) Polynomial regression equations. (**B**) ANOVA and model fit statistics.

(**A**)							
**Regression Equation**							
pH 1.2 *f*_2_ (*Y*_6_) = 40.56 + 1.135 *X*_1_ − 0.206 *X*_2_							
pH 4.0 *f*_2_ (*Y*_10_) = 39.98 + 0.578 *X*_1_ + 0.2207 *X*_2_							
pH 6.8 *f*_2_ (*Y*_14_) = 59.23 + 2.053 *X*_1_ + 0.153 *X*_2_ − 0.1391 *X*_12_							
(**B**)							
**Response Variables**	**Model** **(*p*-Value)**	**Linear Terms** **(*p*-Value)**		**Interaction** **(*p*-Value)**	**Model Fit Statistics (%)**		
		***X*_1_**	***X*_2_**	***X*_1_ *X*_2_**	***R* ^2^**	**Adjusted *R*^2^**	**Predicted *R*^2^**
*Y*_6_	0.010	0.004	0.301	-	60.50	52.60	22.68
*Y*_10_	0.004	0.003	0.042	-	66.55	59.86	49.09
*Y*_14_	0.013	0.035	0.426	-	67.86	57.14	42.22

*R*^2^: coefficient of determination.

**Table 11:** FCCD regression models and ANOVA results for CPP optimization. (**A**) Polynomial regression equations. (**B**) ANOVA and model fit statistics.

(**A**)							
**Regression Equation**							
Friability (*Y*_15_) = 0.9333 − 0.000565 *X*_3_ − 0.0383 *X*_4_ + 0.000000329 *X*_3_^2^							
CI (*Y*_16_) = 41.65 + 0.02375 *X*_3_ − 24.30 *X*_4_ + 6.02 *X*_4_^2^ − 0.00938 *X*_3_*X*_4_							
(**B**)							
**Response Variables**	**Model** **(*p*-Value)**	**Linear Terms** **(*p*-Value)**		**Interaction** **(*p*-Value)**	**Model Fit Statistics (%)**		
		***X*_3_**	***X*_4_**	***X*_3_ *X*_4_**	***R* ^2^**	**Adjusted *R*^2^**	**Predicted *R*^2^**
*Y*_15_	0.001	0.001	0.018	-	82.93	77.24	59.99
*Y*_16_	<0.001	0.029	<0.001	0.004	93.64	90.46	59.96

*R*^2^: coefficient of determination.

**Table 12:** Pharmacokinetic parameters of tirzepatide formulations and benchmark oral semaglutide in beagle dogs.

Formulation	Tirzepatide 0.5 mg	Tirzepatide 14 mg/C10 500 mg	Tirzepatide 14 mg/C10 300 mg	Semaglutide 14 mg (Rybelsus)
Dosing Route	IV	PO	PO	PO
Dose (nmol/kg)	11.61 ± 1.13	428.35 ± 18.92	328.66 ± 39.56	313.46 ± 15.87
*C*_max_ (ng/mL)	-	25.17 ± 5.05	46.49 ± 23.79	26.13 ± 37.56
*T*_max_ (h)	-	1.0 (1.0–1.0)	1.0 (0.5–4.0)	2 (1.0–3.0)
*T*_1/2_ (h)	43.72 ± 7.43	67.4 ± 5.8	57.25 ± 18.77	42.30 ± 24.82
*AUC*_last_ (h⋅ng/mL)	28,257.78 ± 3140.75	790.21 ± 137.45	1261.03 ± 690.44	712.03 ± 995.25

Data are expressed as mean ± SD, except for *T*_max_, which is presented as median (range). IV, intravenous; PO, oral administration; C10, sodium caprate; *AUC*_last_, area under the plasma concentration–time curve from time 0 to the last measurable concentration.

## Data Availability

The original contributions presented in this study are included in the article. Further inquiries can be directed to the corresponding author.

## Supplementary Materials

The following supporting information can be downloaded at: https://www.mdpi.com/article/10.3390/pharmaceutics18070826/s1. Figure S1. Cytotoxicity assessment of C10 and SNAC in Caco-2 cells using MTT and LDH assays; Figure S2: Pareto charts of the standardized effects of critical material attributes (CMAs) on the evaluated responses. The responses are categorized by dissolution medium: pH 1.2 ((A–D): dissolution rates at specific time points), pH 4.0 ((E,F): dissolution rates), and pH 6.8 ((G–I): dissolution rates). The vertical dashed line in each chart indicates the threshold of statistical significance (α = 0.05); Figure S3: Factorial plots of the main and interaction effects of critical material attributes (CMAs) on the evaluated responses. The plots are categorized by dissolution medium: pH 1.2 ((A–D): dissolution rates at specific time points), pH 4.0 ((E,F): dissolution rates), and pH 6.8 ((G–I): dissolution rates; (J): interaction plot for the response shown in panel K); Figure S4: Contour plots showing the effects of critical material attributes (CMAs) on the evaluated responses. The responses are categorized by dissolution medium: pH 1.2 ((A–D): dissolution rates at specific time points), pH 4.0 ((E,F): dissolution rates), and pH 6.8 ((G–I): dissolution rates). The contour lines and color gradients indicate the predicted values of each response; Table S1. Effects of C10 and SNAC on the apparent permeability of tirzepatide across Caco-2 cell monolayers; Table S2. (A) Factor level table and (B) experimental arrangement with evaluated responses for critical material attributes (CMAs); Table S3. Polynomial regression equations for CMA optimization; Table S4. ANOVA and model fit statistics for CMA optimization; Table S5. (A) Factor level table and (B) experimental arrangement with evaluated responses for critical process parameters (CPPs).

## Abbreviations

ANOVA	analysis of variance
*AUC*_last_	area under the plasma concentration–time curve from time zero to the last measurable concentration
BMI	body mass index
C10	sodium caprate
CI	Carr’s index
*C*_max_	maximum plasma concentration
CMAs	critical material attributes
CPPs	critical process parameters
CQAs	critical quality attributes
CROS	crospovidone
DoE	Design of Experiments
DPP-4	dipeptidyl peptidase-4
DS	design space
FCCD	face-centered central composite design
FMEA	failure mode and effects analysis
GIP	glucose-dependent insulinotropic polypeptide
GLP-1	glucagon-like peptide-1
GLP-1 RAs	glucagon-like peptide-1 receptor agonists
HBSS	Hank’s balanced salt solution
HEPES	4-(2-hydroxyethyl)-1-piperazineethanesulfonic acid
HPLC	high-performance liquid chromatography
IACUC	Institutional Animal Care and Use Committee
IV	intravenous
K25	povidone K25
K90	povidone K90
KP	Korean Pharmacopoeia
LDH	lactate dehydrogenase
MRM	multiple reaction monitoring
MRO	multiple-response optimization
MTT	3-(4,5-dimethylthiazol-2-yl)-2,5-diphenyltetrazolium bromide
PET	polyethylene terephthalate
PHA	preliminary hazard analysis
PK	pharmacokinetic
PTFE	polytetrafluoroethylene
QbD	Quality by Design
QRM	quality risk management
QTPP	Quality Target Product Profile
*R* ^2^	coefficient of determination
RPN	risk priority number
RSM	response surface methodology
SC	subcutaneous
SD	standard deviation
SEM	scanning electron microscopy
SNAC	salcaprozate sodium
SSG	sodium starch glycolate
T2DM	type 2 diabetes mellitus
TEER	transepithelial electrical resistance
TFA	trifluoroacetic acid
Tmax	time to reach maximum plasma concentration
*t*_1/2_	elimination half-life
UHPLC–MS/MS	ultra-high-performance liquid chromatography–tandem mass spectrometry
USP	United States Pharmacopeia
VA64	copovidone VA64

## References

[1] Zheng Y., Ley S.H., Hu F.B. (2018). Global aetiology and epidemiology of type 2 diabetes mellitus and its complications. Nat. Rev. Endocrinol..

[2] Phelps N.H., Singleton R.K., Zhou B., Heap R.A., Mishra A., Bennett J.E., Paciorek C.J., Lhoste V.P.F., Carrillo-Larco R.M., Stevens G.A. (2024). Worldwide trends in underweight and obesity from 1990 to 2022: A pooled analysis of 3663 population-representative studies with 222 million children, adolescents, and adults. Lancet.

[3] Narayan K.M.V., Boyle J.P., Thompson T.J., Sorensen S.W., Williamson D.F. (2003). Lifetime Risk for Diabetes Mellitus in the United States. JAMA.

[4] American Diabetes Association Professional Practice Committee (2023). 8. Obesity and Weight Management for the Prevention and Treatment of Type 2 Diabetes: Standards of Care in Diabetes—2024. Diabetes Care.

[5] Davies M.J., Aroda V.R., Collins B.S., Gabbay R.A., Green J., Maruthur N.M., Rosas S.E., Del Prato S., Mathieu C., Mingrone G. (2022). Management of Hyperglycemia in Type 2 Diabetes, 2022. A Consensus Report by the American Diabetes Association (ADA) and the European Association for the Study of Diabetes (EASD). Diabetes Care.

[6] Nauck M.A., Quast D.R., Wefers J., Meier J.J. (2021). GLP-1 receptor agonists in the treatment of type 2 diabetes—State-of-the-art. Mol. Metab..

[7] Kieffer T.J., McIntosh C.H., Pederson R.A. (1995). Degradation of glucose-dependent insulinotropic polypeptide and truncated glucagon-like peptide 1 in vitro and in vivo by dipeptidyl peptidase IV. Endocrinology.

[8] Coskun T., Sloop K.W., Loghin C., Alsina-Fernandez J., Urva S., Bokvist K.B., Cui X., Briere D.A., Cabrera O., Roell W.C. (2018). LY3298176, a novel dual GIP and GLP-1 receptor agonist for the treatment of type 2 diabetes mellitus: From discovery to clinical proof of concept. Mol. Metab..

[9] Jastreboff A.M., Aronne L.J., Ahmad N.N., Wharton S., Connery L., Alves B., Kiyosue A., Zhang S., Liu B., Bunck M.C. (2022). Tirzepatide Once Weekly for the Treatment of Obesity. N. Engl. J. Med..

[10] Rodriguez P.J., Goodwin Cartwright B.M., Gratzl S., Brar R., Baker C., Gluckman T.J., Stucky N.L. (2024). Semaglutide vs Tirzepatide for Weight Loss in Adults with Overweight or Obesity. JAMA Intern. Med..

[11] Mitragotri S., Burke P.A., Langer R. (2014). Overcoming the challenges in administering biopharmaceuticals: Formulation and delivery strategies. Nat. Rev. Drug Discov..

[12] Brayden D.J., Hill T.A., Fairlie D.P., Maher S., Mrsny R.J. (2020). Systemic delivery of peptides by the oral route: Formulation and medicinal chemistry approaches. Adv. Drug Deliv. Rev..

[13] Drucker D.J. (2020). Advances in oral peptide therapeutics. Nat. Rev. Drug Discov..

[14] Homayun B., Lin X., Choi H.-J. (2019). Challenges and Recent Progress in Oral Drug Delivery Systems for Biopharmaceuticals. Pharmaceutics.

[15] Buckley S.T., Bækdal T.A., Vegge A., Maarbjerg S.J., Pyke C., Ahnfelt-Rønne J., Madsen K.G., Schéele S.G., Alanentalo T., Kirk R.K. (2018). Transcellular stomach absorption of a derivatized glucagon-like peptide-1 receptor agonist. Sci. Transl. Med..

[16] Kim D.-H., Kim J.-E. (2025). Recent advances and trends in oral absorption enhancements of GLP-1 receptor agonist formulations. J. Pharm. Investig..

[17] Maher S., Leonard T.W., Jacobsen J., Brayden D.J. (2009). Safety and efficacy of sodium caprate in promoting oral drug absorption: From in vitro to the clinic. Adv. Drug Deliv. Rev..

[18] Krug S.M., Amasheh M., Dittmann I., Christoffel I., Fromm M., Amasheh S. (2013). Sodium caprate as an enhancer of macromolecule permeation across tricellular tight junctions of intestinal cells. Biomaterials.

[19] Halberg I.B., Lyby K., Wassermann K., Heise T., Zijlstra E., Plum-Mörschel L. (2019). Efficacy and safety of oral basal insulin versus subcutaneous insulin glargine in type 2 diabetes: A randomised, double-blind, phase 2 trial. Lancet Diabetes Endocrinol..

[20] Lindmark T., Kimura Y., Artursson P. (1998). Absorption enhancement through intracellular regulation of tight junction permeability by medium chain fatty acids in Caco-2 cells. J. Pharmacol. Exp. Ther..

[21] Kleinebudde P. (2004). Roll compaction/dry granulation: Pharmaceutical applications. Eur. J. Pharm. Biopharm..

[22] Kaerger J.S., Edge S., Price R. (2004). Influence of particle size and shape on flowability and compactibility of binary mixtures of paracetamol and microcrystalline cellulose. Eur. J. Pharm. Sci..

[23] Twarog C., Fattah S., Heade J., Maher S., Fattal E., Brayden D.J. (2019). Intestinal Permeation Enhancers for Oral Delivery of Macromolecules: A Comparison between Salcaprozate Sodium (SNAC) and Sodium Caprate (C10). Pharmaceutics.

[24] Politis S.N., Colombo P., Colombo G., Rekkas D.M. (2017). Design of experiments (DoE) in pharmaceutical development. Drug Dev. Ind. Pharm..

[25] Lee S.-H., Kim J.-E. (2021). Quality by Design Applied Development of Immediate-Release Rabeprazole Sodium Dry-Coated Tablet. Pharmaceutics.

[26] Kim H.-A., Kim J.-E. (2022). Development of Nafamostat Mesylate Immediate-Release Tablet by Drug Repositioning Using Quality-by-Design Approach. Pharmaceutics.

[27] Lee S.-H., Kim J.-K., Jee J.-P., Jang D.-J., Park Y.-J., Kim J.-E. (2022). Quality by Design (QbD) application for the pharmaceutical development process. J. Pharm. Investig..

[28] Son J.-W., Kim H.C., Kim D.-H., Ahn J.-Y., Park Y.-J., Kim J.-E. (2025). Quality-by-design applied development of tianeptine sodium sustained-release once-a-day dosing tablet. J. Pharm. Investig..

[29] Jeon C.-W., Yoon J.-H., Kim J.-E. (2026). QbD-Based Formulation Development of Amiodarone Hydrochloride Tablet. Pharmaceutics.

[30] Artursson P., Palm K., Luthman K. (2001). Caco-2 monolayers in experimental and theoretical predictions of drug transport. Adv. Drug Deliv. Rev..

[31] Yu L.X., Amidon G., Khan M.A., Hoag S.W., Polli J., Raju G.K., Woodcock J. (2014). Understanding pharmaceutical quality by design. AAPS J..

[32] Sjögren E., Abrahamsson B., Augustijns P., Becker D., Bolger M.B., Brewster M., Brouwers J., Flanagan T., Harwood M., Heinen C. (2014). In vivo methods for drug absorption—Comparative physiologies, model selection, correlations with in vitro methods (IVIVC), and applications for formulation/API/excipient characterization including food effects. Eur. J. Pharm. Sci..

[33] Schneider C.A., Rasband W.S., Eliceiri K.W. (2012). NIH Image to ImageJ: 25 years of image analysis. Nat. Methods.

[34] United States Pharmacopeial (2023). <1236> Solubility Measurements. USP-NF.

[35] Bharate S.S., Bharate S.B., Bajaj A.N. (2016). Interactions and incompatibilities of pharmaceutical excipients with active pharmaceutical ingredients: A comprehensive review. Int. J. Pharm. Excip..

[36] Kararli T.T. (1995). Comparison of the gastrointestinal anatomy, physiology, and biochemistry of humans and commonly used laboratory animals. Biopharm. Drug Dispos..

[37] Kim D.-H., Hwang S.-K., Yoon J.-H., Na D.H., Park Y.-J., Chae Y.-J., Chang J.-E., Kim J.-E. (2026). Formulation Engineering of Oral Semaglutide Tablets: Unleashing Gastric Intestinal Permeation with Sodium Caprate. Pharmaceutics.

[38] Mudie D.M., Amidon G.L., Amidon G.E. (2010). Physiological Parameters for Oral Delivery and In Vitro Testing. Mol. Pharm..

[39] Di L. (2015). Strategic approaches to optimizing peptide ADME properties. AAPS J..

[40] Hubatsch I., Ragnarsson E.G.E., Artursson P. (2007). Determination of drug permeability and prediction of drug absorption in Caco-2 monolayers. Nat. Protoc..

[41] Diehl K.H., Hull R., Morton D., Pfister R., Rabemampianina Y., Smith D., Vidal J.M., van de Vorstenbosch C. (2001). A good practice guide to the administration of substances and removal of blood, including routes and volumes. J. Appl. Toxicol..

[42] ICH Expert Working Group (2009). Pharmaceutical Development Q8(R2).

[43] Kang I.-B., Gong S.-J., Kim J.-E. (2026). A Quality-by-Design-Driven Framework for Process Variability Control and Design Space Establishment in Wet Granulation Systems. Processes.

[44] Aroda V.R., Rosenstock J., Terauchi Y., Altuntas Y., Lalic N.M., Morales Villegas E.C., Jeppesen O.K., Christiansen E., Hertz C.L., Haluzík M. (2019). PIONEER 1: Randomized Clinical Trial of the Efficacy and Safety of Oral Semaglutide Monotherapy in Comparison with Placebo in Patients with Type 2 Diabetes. Diabetes Care.

[45] Perez-Gandarillas L., Perez-Gago A., Mazor A., Kleinebudde P., Lecoq O., Michrafy A. (2016). Effect of roll-compaction and milling conditions on granules and tablet properties. Eur. J. Pharm. Biopharm..

[46] Fukuda I.M., Pinto C.F.F., Moreira C.d.S., Saviano A.M., Lourenço F.R. (2018). Design of Experiments (DoE) applied to Pharmaceutical and Analytical Quality by Design (QbD). Braz. J. Pharm. Sci..

[47] Shah R.B., Tawakkul M.A., Khan M.A. (2008). Comparative evaluation of flow for pharmaceutical powders and granules. AAPS PharmSciTech.

[48] United States Pharmacopeial Convention (2023). <616> Bulk Density and Tapped Density of Powders. USP-NF.

[49] United States Pharmacopeial Convention (2023). <1174> Powder Flow. USP-NF.

[50] Ministry of Food and Drug Safety (2022). General Chapter: Bulk Density and Tapped Density of Powders. The Korean Pharmacopoeia.

[51] Carr R.L. (1965). Evaluating flow properties of solids. Chem. Eng..

[52] United States Pharmacopeial Convention (2023). <1216> Tablet Friability. USP-NF.

[53] Ministry of Food and Drug Safety (2022). General Chapter: Tablet Friability Test. The Korean Pharmacopoeia.

[54] United States Pharmacopeial Convention (2023). <711> Dissolution. USP-NF.

[55] Ministry of Food and Drug Safety (2022). General Chapter: Dissolution Test. The Korean Pharmacopoeia.

[56] Ministry of Food and Drug Safety (2022). Regulation on the Standard of Bioequivalence Test for Pharmaceuticals.

[57] Russell W.M.S., Burch R.L. (1959). The Principles of Humane Experimental Technique.

[58] Tannenbaum J., Bennett B.T. (2015). Russell and Burch’s 3Rs then and now: The need for clarity in definition and purpose. J. Am. Assoc. Lab. Anim. Sci..

[59] ICH Expert Working Group (2022). Bioanalytical Method Validation and Study Sample Analysis M10.

[60] Gabrielsson J., Weiner D. (2012). Pharmacokinetic and Pharmacodynamic Data Analysis: Concepts and Applications.

[61] Purves R.D. (1992). Optimum numerical integration methods for estimation of area-under-the-curve (AUC) and area-under-the-moment-curve (AUMC). J. Pharmacokinet. Biopharm..

[62] Montgomery D.C. (2017). Design and Analysis of Experiments.

[63] Derringer G., Suich R. (1980). Simultaneous Optimization of Several Response Variables. J. Qual. Technol..

[64] Aguirre T.A., Teijeiro-Osorio D., Rosa M., Coulter I.S., Alonso M.J., Brayden D.J. (2016). Current status of selected oral peptide technologies in advanced preclinical development and in clinical trials. Adv. Drug Deliv. Rev..

[65] Serajuddin A.T., Thakur A.B., Ghoshal R.N., Fakes M.G., Ranadive S.A., Morris K.R., Varia S.A. (1999). Selection of solid dosage form composition through drug-excipient compatibility testing. J. Pharm. Sci..

[66] Kalantzi L., Goumas K., Kalioras V., Abrahamsson B., Dressman J.B., Reppas C. (2006). Characterization of the human upper gastrointestinal contents under conditions simulating bioavailability/bioequivalence studies. Pharm. Res..

[67] ICH Expert Working Group (2023). Quality Risk Management Q9(R1).

[68] Djuris J., Medarevic D., Krstic M., Djuric Z., Ibric S. (2013). Application of quality by design concepts in the development of fluidized bed granulation and tableting processes. J. Pharm. Sci..

[69] Montgomery D.C., Peck E.A., Vining G.G. (2012). Introduction to Linear Regression Analysis.

[70] Charoo N.A., Shamsher A.A., Zidan A.S., Rahman Z. (2012). Quality by design approach for formulation development: A case study of dispersible tablets. Int. J. Pharm..

[71] George C. (1994). A Balancing Act: Optimizing a Product’s Properties. Qual. Prog..

[72] Qiu Y., Chen Y., Zhang G.G., Yu L., Mantri R.V. (2016). Developing Solid Oral Dosage Forms: Pharmaceutical Theory and Practice.

[73] Box G.E.P., Hunter J.S., Hunter W.G. (2005). Statistics for Experimenters: Design, Innovation, and Discovery.

[74] Kikuta J.-I., Kitamori N. (1994). Effect of Mixing Time on the Lubricating Properties of Magnesium Stearate and the Final Characteristics of the Compressed Tablets. Drug Dev. Ind. Pharm..

[75] United States Pharmacopeial Convention (2023). USP General Chapter <1174> Powder Flow, and <1216> Tablet Friability.

[76] Bezerra M.A., Santelli R.E., Oliveira E.P., Villar L.S., Escaleira L.A. (2008). Response surface methodology (RSM) as a tool for optimization in analytical chemistry. Talanta.

[77] Lionberger R.A., Lee S.L., Lee L., Raw A., Yu L.X. (2008). Quality by design: Concepts for ANDAs. AAPS J..

[78] Moore J.W., Flanner H.H. (1996). Mathematical comparison of dissolution profiles. Pharm. Technol..

[79] Cowart K. (2020). Oral Semaglutide: First-in-Class Oral GLP-1 Receptor Agonist for the Treatment of Type 2 Diabetes Mellitus. Ann. Pharmacother..

[80] Levin M. (2001). Pharmaceutical Process Scale-Up.

[81] Granhall C., Donsmark M., Blicher T.M., Golor G., Søndergaard F.L., Thomsen M., Bækdal T.A. (2019). Safety and Pharmacokinetics of Single and Multiple Ascending Doses of the Novel Oral Human GLP-1 Analogue, Oral Semaglutide, in Healthy Subjects and Subjects with Type 2 Diabetes. Clin. Pharmacokinet..

