# Supplementary material for: Overcoming Gastric Barriers for Oral Peptide Delivery: QbD-Based Development of Sodium Caprate-Enabled Tirzepatide Tablets
Source: Pharmaceutics. 2026 Jul 5;18(7):826. doi: 10.3390/pharmaceutics18070826 (PMC13415043; doi:10.3390/pharmaceutics18070826)
Supplement: Supplementary file 1 [file pharmaceutics-18-00826-s001.zip › Supplementary Tables.pdf]

Table S1. Effects of C10 and SNAC on the apparent permeability of tirzepatide across Caco-2 cell monolayers.

Permeation enhancer	*P*app (cm/s)	Fold change
Control (Tirzepatide alone)	4.49×10^{-7}	-
C10 2.5 mM	4.63×10^{-7}	1.0
C10 5 mM	9.01×10^{-7}	2.0
C10 10 mM	1.84×10^{-6}	4.1
SNAC 10 mM	9.78×10^{-7}	2.2
SNAC 20 mM	1.53×10^{-6}	3.4

Table S2. (A) Factor level table and (B) experimental arrangement with evaluated responses for critical material attributes (CMAs).

(A)

**Factor**	**Unit**	**Low (-1)**	**Medium (0)**	**High (+1)**
X_1: Binder ratio (**Povidone K90** : Copovidone)	mg	(**0**:20)	(**10**:10)	(**20**:0)
X_2: Disintegrant ratio (**SSG** : Crospovidone)	mg	(**0**:32)	(**16**:16)	(**32**:0)

(B)

**Run**	X_1	X_2	Y_1	Y_2	Y_3	Y_4	Y_5	Y_6	Y_7	Y_8	Y_9	Y_{10}	Y_{11}	Y_{12}	Y_{13}	Y_{14}
1	0	32	37	54	85	96	98	44.05	54	88	97	48.96	39	65	96	63.46
2	10	0	28	42	73	86	92	68.1	54	86	98	50.83	46	72	105	68.77
3	10	16	35	51	83	95	98	46.2	56	88	99	47.58	36	56	101	52.47
4	10	32	36	53	87	97	99	42.81	52	86	98	52.54	43	70	102	72.94
5	20	0	26	41	72	88	94	62.4	54	86	99	50.06	35	59	90	49.42
6	10	16	35	50	81	96	101	45.99	51	88	99	51.36	41	67	97	69.68
7	20	16	24	37	68	85	93	63.77	51	84	97	55.51	30	54	86	41.29
8	10	16	37	53	79	96	100	46.04	60	94	101	40.09	42	66	98	73.91
9	20	32	27	41	73	91	96	57.1	48	82	98	61.96	33	60	94	50.56
10	0	16	44	62	90	97	100	37.91	59	91	99	42.54	41	72	102	67.45
11	10	16	37	53	84	97	99	43.97	54	87	99	49.09	50	74	103	65.52
12	10	16	38	55	86	100	102	40.47	55	87	97	48.78	47	70	104	74.76

13	0	0	50	69	95	98	100	33.21	60	92	100	41.38	49	76	111	54.11

X_1 and X_2 represent the weight ratios of Povidone K90 to Copovidone and sodium starch glycolate (SSG) to Crospovidone, respectively (bolded numbers indicate the actual factor levels inputted into the model). The total amounts of the binder and disintegrant mixtures were kept constant at 20 mg and 32 mg per tablet, respectively. The evaluated responses (Y_1-Y_{14}) encompass dissolution rates at specific time points and similarity factors (f_2) across three pH conditions: pH 1.2 (10, 15, 30, 45, and 60 min for Y_1-Y_5; f_2 for Y_6), pH 4.0 (10, 20, and 30 min for Y_7-Y_9; f_2 for Y_{10}), and pH 6.8 (10, 15, and 30 min for Y_{11}-Y_{13}; f_2 for Y_{14}).

Table S3. Polynomial regression equations for CMA optimization..

**Regression equation**
pH1.2 10min (Y_1) = 44.59 - 0.900 X_1 - 0.042 X_2
pH1.2 15min (Y_2) = 62.51 - 1.100 X_1 - 0.042 X_2
pH1.2 30min (Y_3) = 89.90 - 0.950 X_1 + 0.052 X_2
pH1.2 45min (Y_4) = 96.50 - 0.450 X_1 + 0.1250 X_2
pH1.2 60min (Y_5) = 99.18 - 0.250 X_1 + 0.0729 X_2
pH4.0 10min (Y_7) = 60.13 - 0.3333 X_1 - 0.1458 X_2
pH4.0 20min (Y_8) = 92.12 - 0.3167 X_1 - 0.0833 X_2
pH4.0 30min (Y_9) = 99.538 - 0.0333 X_1 - 0.0417 X_2
pH6.8 10min (Y_{11}) = 45.50 + 0.631 X_1 - 0.156 X_2 - 0.0574 X_1^2
pH6.8 15min (Y_{12}) = 74.90 - 0.667 X_1 - 0.125 X_2
pH6.8 30min (Y_{13}) = 110.08 - 0.139 X_1 - 0.443 X_2 - 0.0493 X_1^2 + 0.02969 X_1X_2

Table S4. ANOVA and model fit statistics for CMA optimization.

Response variables	Model (p-value)	Linear terms (p-value)		Interaction (p-value)	Model fit statistics (%)		
		X_1	X_2	X_1X_2	R^2	Adjusted R^2	Predicted R^2
Y_1	0.001	< 0.001	0.686	-	76.01	71.21	47.09
Y_2	0.001	< 0.001	0.741	-	75.93	71.11	48.14
Y_3	0.001	< 0.001	0.660	-	72.92	67.50	45.00
Y_4	0.027	0.014	0.214	-	51.60	41.91	17.20
Y_5	0.090	0.048	0.318	-	38.15	25.78	0.00
Y_7	0.007	0.007	0.039	-	63.18	55.81	50.06
Y_8	0.015	0.008	0.191	-	56.63	47.96	40.33
Y_9	0.341	0.504	0.196	-	19.35	3.21	0.00
Y_{11}	0.017	0.014	0.176	-	65.96	54.62	38.02
Y_{12}	0.036	0.015	0.398	-	48.58	38.30	18.25
Y_{13}	0.002	0.001	0.102	0.015	85.56	78.33	50.80

Table S5. (A) Factor level table and (B) experimental arrangement with evaluated responses for critical process parameters (CPPs).

(A)

Factor	Unit	Low (-1)	Medium (0)	High (+1)
X_3: Rotation speed	rpm	200	600	1000
X_4: Sieve size	mm	1	2	3

(B)

Run	Milling process		Particle size distribution (%)						Responses	
	Rotation speed (rpm, X_3)	Sieve size (mm, X_4)	> 1410 µm	841 µm	500 µm	355 µm	74 µm	< 74 µm	Friability (%, Y_{15})	Carr's Index (Y_{16})
1	1000	3	2	12	31	14	36	4	0.59	20
2	200	1	0	0	31	19	47	3	0.81	26
3	600	2	0	9	55	9	25	1	0.67	19
4	600	2	0	11	56	8	23	1	0.65	20
5	600	3	7	27	28	10	23	6	0.57	20
6	600	2	0	9	54	10	25	1	0.66	20
7	600	1	0	0	15	31	50	4	0.63	30
8	1000	2	0	4	44	17	33	2	0.58	20
9	200	3	41	25	12	5	14	3	0.74	21
10	600	2	0	10	54	9	25	1	0.64	20
11	200	2	9	45	19	7	20	1	0.72	21
12	1000	1	0	0	4	26	58	12	0.69	40
13	600	2	0	9	54	10	24	1	0.63	21
