# Supplementary material for: Overcoming Gastric Barriers for Oral Peptide Delivery: QbD-Based Development of Sodium Caprate-Enabled Tirzepatide Tablets
Source: Pharmaceutics. 2026 Jul 5;18(7):826. doi: 10.3390/pharmaceutics18070826 (PMC13415043; doi:10.3390/pharmaceutics18070826)
Supplement: Supplementary file 1 [file pharmaceutics-18-00826-s001.zip › Supplementary Figures.pdf]

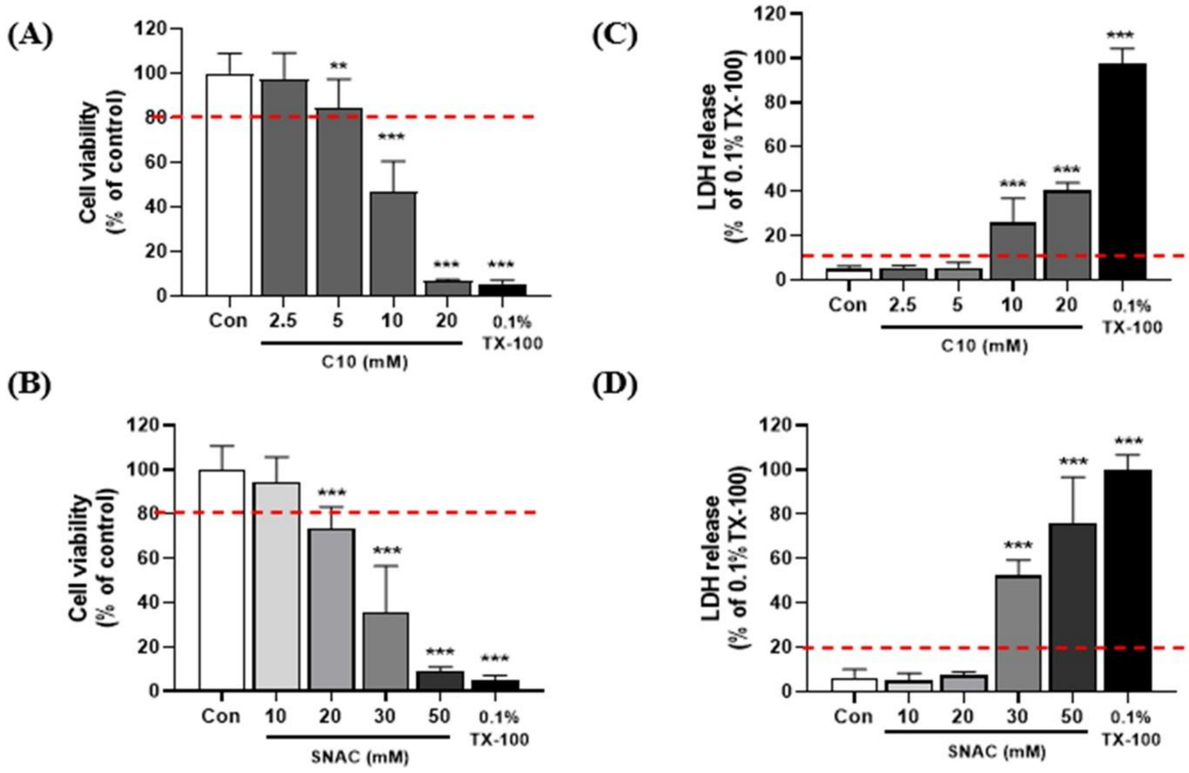

Figure S1. Cytotoxicity assessment of C10 and SNAC in Caco-2 cells using MTT and LDH assays. Cell viability was evaluated by MTT assay after treatment with (A) C10 and (B) SNAC. Membrane integrity was assessed by LDH release assay after treatment with (C) C10 and (D) SNAC. Triton X-100 (0.1%) was used as the positive control. Data are expressed as mean ± SD ($n = 3$). $^{**}p < 0.01$ and $^{***}p < 0.001$ versus control.

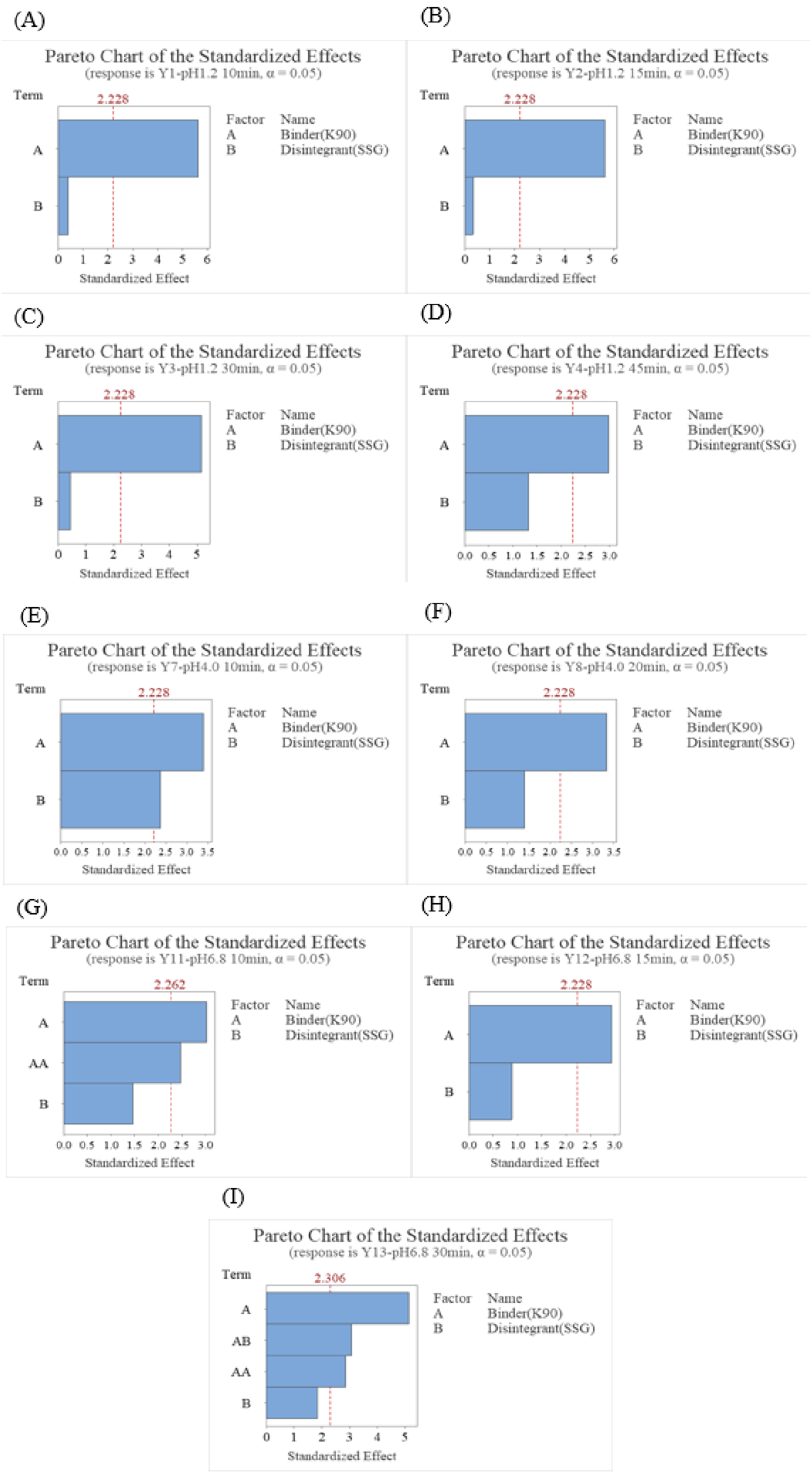

Figure S2. Pareto charts of the standardized effects of critical material attributes (CMAs) on the evaluated responses. The responses are categorized by dissolution medium: pH 1.2 (A–D: dissolution rates at specific time points), pH 4.0 (E–F: dissolution rates), and pH 6.8 (G–I: dissolution rates). The vertical dashed line in each chart indicates the threshold of statistical significance (α = 0.05).

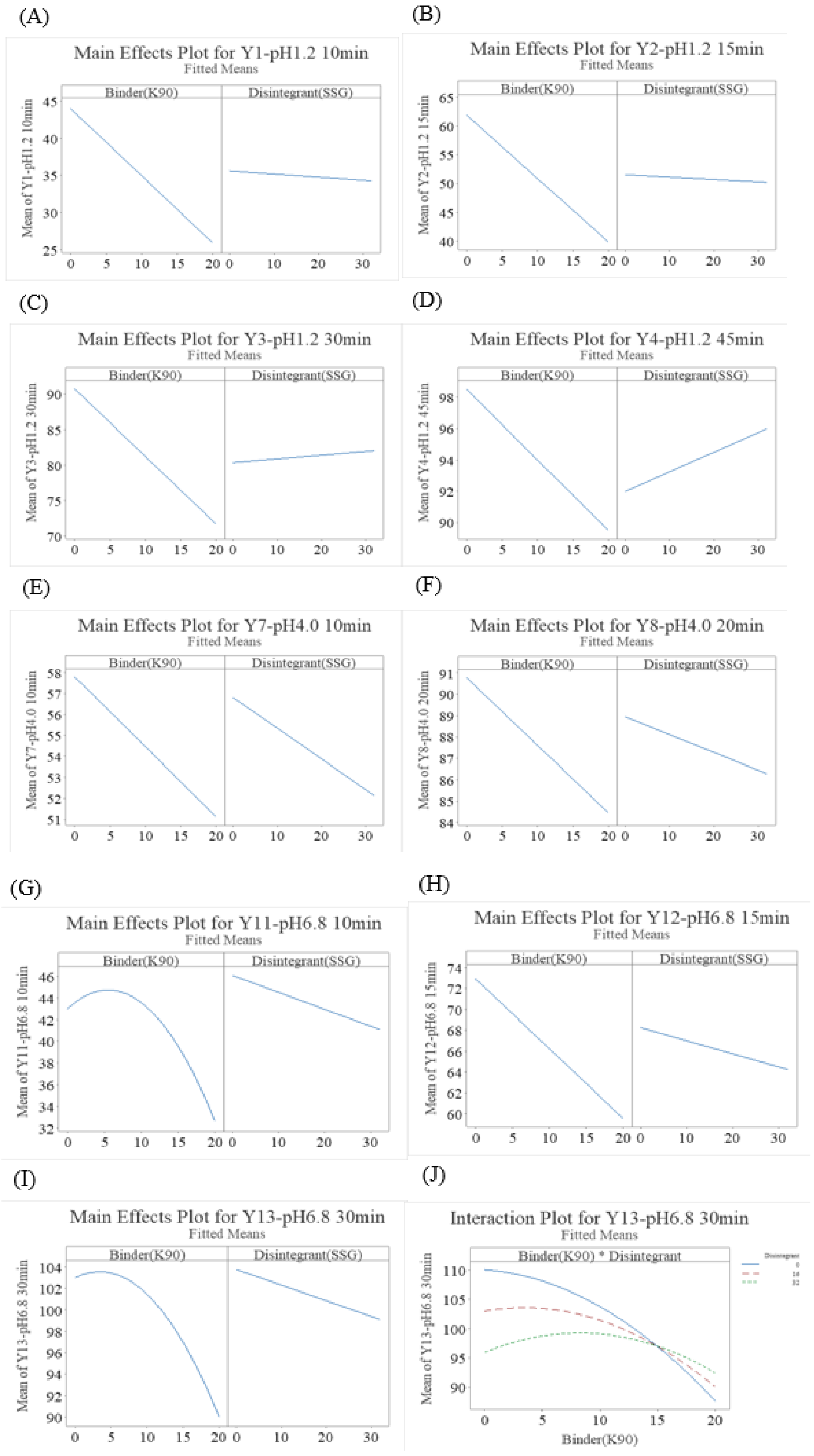

Figure S3. Factorial plots of the main and interaction effects of critical material attributes (CMAs) on the evaluated responses. The plots are categorized by dissolution medium: pH 1.2 (A–D: dissolution rates at specific time points), pH 4.0 (E–F: dissolution rates), and pH 6.8 (G–I: dissolution rates; J: interaction plot for the response shown in panel K).

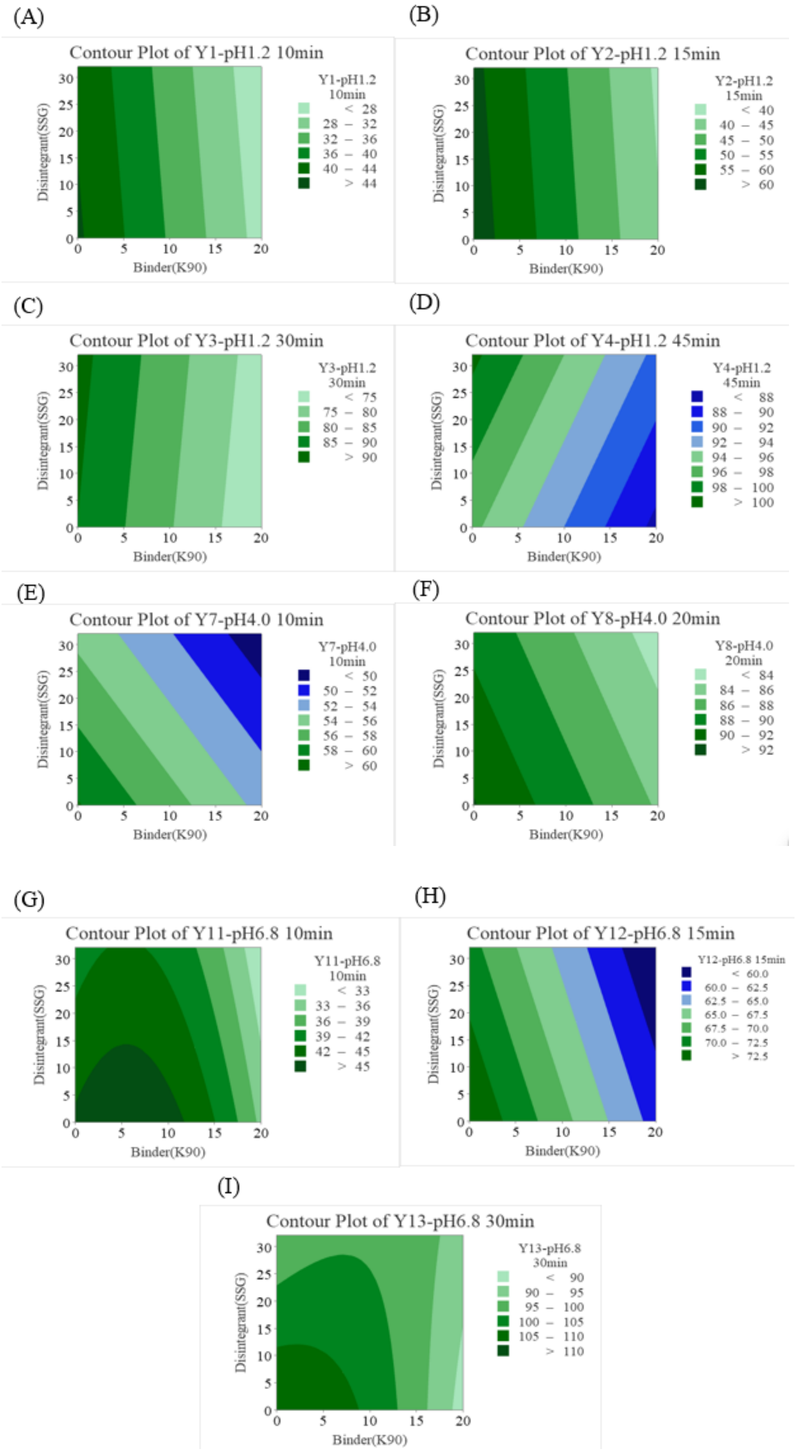

Figure S4. Contour plots showing the effects of critical material attributes (CMAs) on the evaluated responses. The responses are categorized by dissolution medium: pH 1.2 (A–D: dissolution rates at specific time points), pH 4.0 (E–F: dissolution rates), and pH 6.8 (G–I: dissolution rates). The contour lines and color gradients indicate the predicted values of each response.
